# The Simultaneous Prevention of Multiple Diseases: A “One Ring to Rule Them All” Framework for Redox-Driven Health and Longevity

**DOI:** 10.3390/nu18061007

**Published:** 2026-03-22

**Authors:** Harold Robert Silverstein, Albert A. Rizvanov, Donald David Haines, Fadia F. Mahmoud, Stephen Christopher Rose, Valeriya V. Solovyeva, Kristina V. Kitaeva, Arpad Tosaki

**Affiliations:** 1 Preventive Medicine Center (PMC), 1000 Asylum Avenue #2109, Hartford, CT 06105, USA; h.robert.silverstein@gmail.com; 2 Institute of Fundamental Medicine and Biology, Kazan (Volga Region) Federal University, 420008 Kazan, Russia; solovyovavv@gmail.com (V.V.S.); kitaeva.kristina.v@gmail.com (K.V.K.); 3 Division of Medical and Biological Sciences, Tatarstan Academy of Sciences, 420111 Kazan, Russia; 4 Advanced Biotherapeutics Ltd., 20–22 Wenlock Road, London N1 7GU, UK; donalbain88@yahoo.com (D.D.H.); fadia.frcpath@gmail.com (F.F.M.); 5 College of Nanotechnology, Science, and Engineering, SUNY Albany, Albany, NY 12222, USA; chris@geronimo.life; 6 Department Pharmacology, Faculty of Pharmacy, University of Debrecen, Nagyerdei krt. 98, 4032 Debrecen, Hungary; 7 HUN-REN-UD Pharmamodul Research Group, Faculty of Pharmacy, University of Debrecen, Nagyerdei krt. 98, 4032 Debrecen, Hungary

**Keywords:** antioxidants, oxidative stress, inflammation, cardiovascular aging, lifestyle medicine, senescence, SASP, mTOR, AMPK, microbiome, TMAO, nutraceuticals, gene therapy, orphan diseases, longevity

## Abstract

Chronic non-communicable diseases rarely occur in isolation; cardiovascular, metabolic, neurodegenerative, malignant, and age-associated disorders share upstream drivers including oxidative stress, chronic inflammation, mitochondrial dysfunction, and metabolic imbalance. This narrative review synthesizes epidemiological, interventional, and mechanistic studies identified through targeted literature searches to examine redox biology as a shared mechanistic hub linking these conditions. We evaluate antioxidant-rich dietary patterns, selected nutraceuticals, myocardial ischemia–reperfusion injury as a clinical exemplar, rare redox-imbalance disorders as mechanistic stress models, and emerging gene-based reinforcement of endogenous antioxidant systems. Rather than proposing clinical targets, we present an integrative, hypothesis-generating framework illustrating how coordinated lifestyle-driven modulation of redox balance may simultaneously influence multiple disease trajectories. Collectively, the evidence supports a unified redox framework for multi-disease prevention for multi-disease prevention and future intervention design.

## 1. Introduction

Chronic non-communicable diseases rarely occur in isolation. Cardiovascular disease, type 2 diabetes, cancer, neurodegenerative disorders, and other age-associated conditions (we have combined them into the term “multiple diseases”) share overlapping biological drivers, including oxidative stress, chronic low-grade inflammation, metabolic dysregulation, mitochondrial dysfunction, and cellular senescence. Although cardiovascular disease remains a leading global cause of mortality [1], it represents only one manifestation of a broader, systemic redox-inflammatory imbalance that contributes to multiple chronic pathologies. Rather than viewing these disorders as independent entities, increasing evidence supports a unified mechanistic perspective in which shared upstream processes give rise to diverse clinical outcomes.

Beyond mortality and morbidity, the economic burden of chronic non-communicable diseases is substantial. In the U.S. alone, healthcare expenditures related to cardiovascular disease exceed USD 400 billion annually, and total direct and indirect costs of cardiometabolic diseases account for approximately 20% of national healthcare spending [2,3]. Globally, the economic impact of chronic diseases continues to rise, reinforcing the need for scalable preventive strategies targeting shared upstream mechanisms such as oxidative stress and chronic inflammation [4].

While there is a genetic predisposition to most chronic illnesses, lifestyle factors—particularly dietary choices—are the major determinants that lead to the expression of those diseases. Other key factors include breathing (air quality and smoking), drinking (hydration and alcohol use), physical activity, and psychological stress. In practical terms, these can be described as the five basic health activities: eating, breathing, drinking, exercise, and mental well-being. Each corresponds to modifiable behaviors: consuming a predominantly whole-food, plant-based diet; avoiding smoking and air pollutants; maintaining adequate hydration while limiting alcohol and sugary beverages; engaging in regular exercise; and managing stress with healthy social and psychological practices. Optimization of these five lifestyle domains has broad preventive effects—for example, comprehensive lifestyle programs have demonstrated simultaneous improvements in cardiovascular and cancer outcomes [5]—supporting the concept that “the triggers are lifestyle choices” even if “genes load the gun”.

Cardiovascular disease provides a particularly well-characterized clinical model through which redox-dependent mechanisms can be examined. However, the same biological pathways that drive atherosclerosis and ischemia-reperfusion (I/R) injury are also implicated in metabolic syndrome, neurodegeneration, oncogenesis, and frailty. Beyond behaviors, specific “wellness-protecting” clinical targets can be identified. Attaining certain quantifiable health metrics has been associated with dramatic reductions in disease risk. For instance, long-term exposure to low low-density lipoprotein (LDL) cholesterol (e.g., ~70–80 mg/dL) is associated with substantially lower risk of atherosclerotic cardiovascular events across genetic, epidemiologic, and randomized-trial evidence [6,7,8,9]. Landmark lifestyle trials showed that intensive dietary and habit changes can not only prevent but also reverse coronary artery disease—patients in the Lifestyle Heart Trial who adopted a plant-based diet with exercise and stress management actually exhibited regression of coronary lesions [10]. Likewise, maintaining blood pressure in the normal range (e.g., <120/80 mmHg) is a crucial goal; home monitoring and proactive management of blood pressure have been advocated to help individuals achieve and sustain control and reduce cardiovascular risk [7,11]. These examples illustrate that hitting “protective” numbers for cholesterol, blood pressure, and other risk factors—largely through natural lifestyle means or adjunct therapies—can profoundly limit the manifestation of disease.

Crucially, many disease-generating pathways can be attenuated or even arrested by early interventions, lowering the risk of diseases before they ever manifest. Epidemiological analyses suggest that a substantial proportion of atherosclerotic, diabetic, and hypertensive conditions may be attributable to modifiable lifestyle factors, particularly among genetically predisposed individuals; however, precise estimates vary across populations and study designs. Even when such conditions are present, comprehensive lifestyle changes often can stabilize or partially reverse them for all practical purposes [10,12]. Some traditional or less industrialized populations historically demonstrated markedly lower prevalence of certain chronic illnesses compared to highly industrialized societies, although such comparisons are influenced by demographic, environmental, and diagnostic differences. Heritage diets and behaviors in certain traditional or agrarian cultures do not trigger those genetic predispositions—for example, when individuals adopt a high-fiber, plant-rich diet typical of some African heritage populations, inflammatory and metabolic risk markers improve markedly compared to a Western diet [13]. In other words, the Western lifestyle “pulls the trigger” on genes that would otherwise remain silent. There are effective lifestyle deterrents to many—if not most—of the costly chronic conditions that hound modern societies, which currently consume on the order of one-fifth of the U.S. economy in healthcare expenditures [14].

At the biological level, unhealthy behaviors such as excessive caloric intake (leading to obesity or elevated abdominal fat) and poor diet quality activate pro-aging and pro-growth signaling pathways with inappropriate immune activation. In particular, nutrient excess and visceral adiposity tilt the balance away from the housekeeping, survival, and repair pathway, AMP-activated protein kinase (AMPK), and toward the growth-promoting kinase, mechanistic target of rapamycin (mTOR) [15]. This mTOR predominance drives cellular proliferation and inhibits autophagy, contributing to the accumulation of damaged cellular components and a chronic inflammatory state. Meanwhile, inadequate energy balance (and lack of hormetic stress from exercise or fasting) means reduced AMPK activity and impaired cellular repair. Reactive oxygen species (ROS)—the “dangerous” reactive forms of oxygen—along with unbalanced, pro-inflammatory cytokines, contribute to a self-reinforcing cascade of oxidative and inflammatory signaling in human tissues. Oxidative damage to macromolecules and the overzealous inflammatory and growth signals of aging (sometimes termed “inflammaging”) drive tissue dysfunction [15,16]. Below, we address these processes with specific examples.

### 1.1. Objective and Novelty

Prior reviews commonly address antioxidants within a single disease silo (e.g., cardiovascular disease alone) or focus on isolated supplements without an integrative model linking shared upstream biology to multi-disease outcomes. The objective of this narrative review is to synthesize evidence that redox imbalance—driven by metabolic overload, mitochondrial dysfunction, and downstream inflammatory and senescence pathways—constitutes a shared mechanistic hub across cardiometabolic, malignant, neurodegenerative, and other age-associated conditions, and to map scalable lifestyle and dietary antioxidant strategies onto that hub. We further use myocardial I/R injury as a clinically relevant “stress test” of ROS-driven pathology and briefly discuss rare (orphan) diseases and gene-based reinforcement of endogenous antioxidant defenses as translational edge cases and future directions. The review is organized into eight sections covering shared mechanisms, lifestyle and dietary patterns, targeted nutraceuticals with a cardiovascular exemplar, orphan disease lessons, gene-based concepts, and an integrative framework (Table 1).

### 1.2. Literature Identification and Scope

This is a narrative review. We searched PubMed and Google Scholar using combinations of terms including oxidative stress, redox, antioxidants, polyphenols, dietary patterns, Mediterranean diet, fiber, microbiome, trimethylamine N-oxide (TMAO), senescence, senescence-associated secretory phenotype (SASP), mTOR, AMPK, ischemia reperfusion, cardioprotection, lysosomal storage disease (LSD), and gene therapy antioxidant enzymes. We prioritized systematic reviews, randomized trials, major cohort studies, and well-established mechanistic studies; additional disease-model and proof-of-concept gene-delivery studies were included where they clarified redox biology relevant to the Special Issue theme.

## 2. Shared Redox Mechanisms Underlying Multiple Diseases

Importantly, the mechanistic pathways described below are not isolated biochemical events but represent nodes that are modifiable through dietary and lifestyle exposures discussed in later sections. For example, mTOR predominance may be influenced by caloric excess, whereas AMPK activation is promoted by exercise and caloric moderation; mitochondrial ROS generation may be attenuated by polyphenol-induced nuclear factor erythroid 2-related factor 2 (Nrf2) activation; and inflammatory amplification can be modulated by fiber-driven microbiome metabolites. Thus, the mechanistic primer in this section provides the biological foundation for the intervention strategies discussed subsequently.

**Figure 1:**
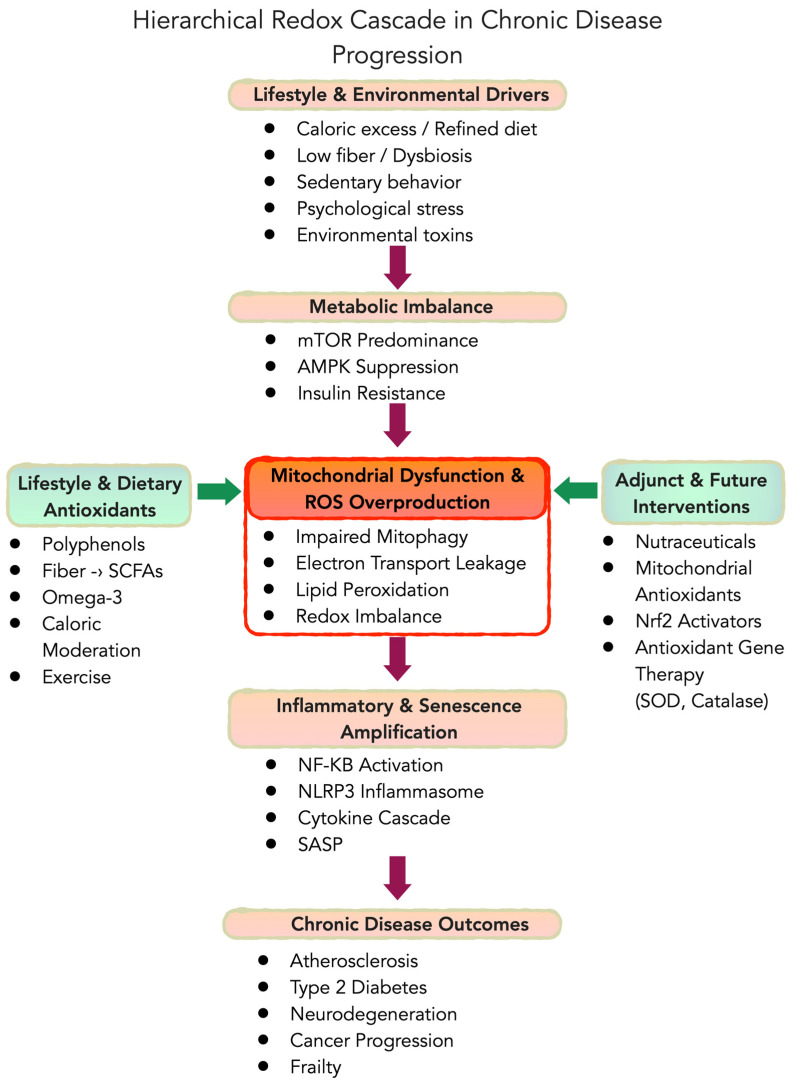
Hierarchical redox cascade linking upstream lifestyle and metabolic drivers to multi-organ chronic disease outcomes. Caloric excess, dysbiosis, physical inactivity, and stress promote mTOR predominance, mitochondrial dysfunction, and excess ROS production. Elevated ROS amplifies inflammatory signaling (e.g., NF-κB activation), cellular senescence, and SASP-mediated tissue dysfunction, ultimately contributing to cardiovascular, metabolic, neurodegenerative, and oncological diseases. Lifestyle interventions and dietary antioxidants primarily act upstream and at the level of mitochondrial redox balance, whereas targeted nutraceuticals and emerging gene-based antioxidant strategies may modulate downstream amplification pathways. The schematic reflects a literature-informed mechanistic synthesis rather than a quantitative causal model.

Although oxidative stress, inflammation, metabolic dysregulation, mitochondrial dysfunction, and cellular senescence interact bidirectionally, a hierarchical heuristic provides conceptual clarity (Figure 1). In this framework, upstream lifestyle and metabolic drivers (e.g., caloric excess, nutrient imbalance, physical inactivity) promote mTOR predominance and suppression of AMPK signaling, leading to mitochondrial dysfunction and excess ROS generation. Elevated ROS then amplify inflammatory cascades (e.g., nuclear factor kappa-light-chain-enhancer of activated B cells (NF-κB) activation) and promote cellular senescence and SASP, which collectively drive tissue dysfunction and chronic disease. The microbiome acts as a dynamic modulator of this cascade, capable of either amplifying oxidative stress (e.g., via TMAO production) or reinforcing redox resilience (e.g., via short-chain fatty acids and urolithins).

### 2.1. Oxidative Stress and Chronic Low-Grade Inflammation

Oxidative stress reflects an imbalance between oxidant generation and antioxidant defenses, resulting in oxidative modification of lipids, proteins, and nucleic acids in tissues [29]. Persistent oxidative stress promotes and amplifies chronic low-grade inflammation through redox-sensitive signaling, including activation of NF-κB and other transcriptional programs [30]. In turn, inflammation can further increase ROS generation via immune-cell activation and mitochondrial dysfunction, creating a self-reinforcing loop of oxidative damage and inflammatory signaling that is relevant across cardiovascular, metabolic, neurodegenerative, and malignant diseases. Indeed, oxidative stress and inflammation are tightly intertwined drivers of pathology in virtually all age-related chronic diseases [16,31]. This bidirectional oxidative-inflammation cycle is now understood as a common soil for disease development.

### 2.2. Metabolic Dysregulation and Insulin Resistance

Metabolic dysregulation represents an upstream driver in this cascade (Figure 1). Metabolic syndrome and insulin resistance are tightly linked to redox imbalance [32,33]. Nutrient overload, ectopic fat deposition, and mitochondrial stress all increase ROS production and activate inflammatory pathways, contributing to endothelial dysfunction and accelerated vascular aging. This metabolic–redox coupling helps explain why cardiometabolic interventions often produce broad “multi-disease” benefits: improvements in diet, body composition, and exercise can simultaneously reduce risk of diabetes, cardiovascular disease, and certain cancers [5]. For example, visceral adiposity is not merely a passive risk factor—it actively secretes pro-inflammatory adipokines that contribute to chronic heart failure and other conditions (the “adipokine hypothesis” of heart failure) [34]. By the same token, weight loss, caloric moderation, and fasting-mimicking diets reduce oxidative burden and inflammation, yielding system-wide benefits. The upshot is that ameliorating metabolic stress through lifestyle changes or targeted therapies can concurrently delay or prevent multiple diseases driven by those same redox and inflammatory disturbances.

### 2.3. Cellular Senescence and the SASP

Cellular senescence and SASP represent downstream amplifiers of prolonged redox and inflammatory stress rather than primary initiating events. Senescent cells secrete a pro-inflammatory mix of cytokines, chemokines, growth factors, and proteases known as the SASP, which can drive tissue dysfunction and propagate inflammation in neighboring cells [35,36]. Oxidative stress is both a trigger and a consequence of cellular senescence, placing the senescence/SASP phenomenon near the center of multi-disease aging biology. In fact, accumulating oxidative damage with age leads to hyperactivity of pro-growth pathways like mTOR, impaired proteostasis (e.g., buildup of lipofuscin and misfolded proteins), and a feedback loop of further ROS generation and SASP factor release [15]. Senescent cells effectively poison their microenvironment—for example, senescent endothelial cells reduce nitric oxide and promote inflammation in blood vessels, contributing to atherosclerosis. Removing senescent cells (in animal models) leads to delayed aging and preserved organ function, highlighting their pathological role. Therapies targeting senescent cells (senolytics) or suppressing the SASP are being explored as multi-disease preventive strategies [15].

### 2.4. mTOR/AMPK Balance, Autophagy, and Mitochondrial Function

At the apex of nutrient-sensitive regulation, the mTOR/AMPK balance acts as a gatekeeper determining whether cells shift toward growth-promoting or repair-oriented states. The mTOR–AMPK axis acts as a central “growth vs. repair” switch linking nutrient availability to an organism’s redox state and aging rate [15]. Chronic nutrient excess and anabolic signals favor mTOR activation (promoting growth and cell proliferation), while energy stress (e.g., calorie restriction or exercise) activates AMPK (promoting maintenance, autophagy, and efficient metabolism). Autophagy—the process by which cells remove or recycle damaged components, including dysfunctional mitochondria (mitophagy)—is crucial for limiting oxidative damage. If autophagy is impaired (as occurs with aging and persistent mTOR activation), damaged mitochondria accumulate and produce more ROS, further activating inflammatory pathways. Thus, interventions that tip the balance toward AMPK activation and robust autophagy (such as caloric restriction mimetics, regular exercise, or compounds like metformin) may confer multi-system benefits by reducing chronic oxidative stress. Indeed, AMPK activation has been shown to extend healthspan in multiple model organisms, whereas hyperactive mTOR accelerates aging processes [15,17,18]. This mechanistic insight underlies the growing interest in lifestyle and pharmacological strategies that modulate mTOR/AMPK—for instance, fasting regimes or mTOR inhibitors (like rapamycin)—as potential broad-spectrum disease-preventive approaches.

### 2.5. Microbiome-Driven Inflammation and TMAO

The gut microbiome is an increasingly recognized regulator of systemic inflammation and redox status through its metabolic products and its influence on host pathways. A notable example is TMAO, a pro-atherogenic molecule produced when gut bacteria metabolize dietary choline/carnitine (from red meat, eggs, etc.), with the intermediate trimethylamine oxidized in the liver. Elevated TMAO has been associated with increased cardiometabolic risk and vascular dysfunction in both animals and humans [19]. Diets high in fiber and polyphenols (e.g., plant-rich diets) support a more favorable gut microbiota, increasing the production of beneficial metabolites (like short-chain fatty acids) while reducing TMAO levels and other harmful compounds [19]. Indeed, switching individuals from a Western diet to a high-fiber, plant-focused diet can rapidly alter microbial composition and lower inflammatory tone [13]. Conversely, dysbiosis and increased gut permeability can lead to greater translocation of endotoxins and activation of systemic inflammation. Thus, the microbiome represents a key mediator between diet and the host’s redox-inflammatory state [19]. Interventions ranging from probiotics and prebiotic fibers to TMAO-lowering strategies are being investigated to harness the microbiome for chronic disease prevention. Notably, the microbiome’s impact is not limited to the gut or cardiovascular system—it also influences cardiometabolic risk and cancer biology via immune and metabolic modulation [19,37]. Managing diet quality to cultivate a healthy microbiome is, therefore, a critical piece of the multi-disease prevention puzzle.

Beyond pro-atherogenic metabolites such as TMAO, diet–microbiome interactions also generate metabolites with potentially protective redox effects. High-fiber and polyphenol-rich dietary patterns promote the expansion of short-chain fatty acid (SCFA)-producing taxa, including butyrate-generating bacteria, which have been shown to improve epithelial barrier integrity, reduce systemic inflammation, and modulate mitochondrial function [38,39]. SCFAs such as butyrate can influence histone acetylation and AMP-activated protein kinase signaling, thereby linking microbial fermentation products to host redox regulation and metabolic homeostasis [16].

In addition, gut microbial metabolism of dietary ellagitannins and other polyphenols generates urolithins, which have been associated with enhanced mitophagy and improved mitochondrial function in experimental models [40]. These interactions suggest a reinforcing loop: antioxidant-rich dietary patterns shape the microbiome toward metabolite profiles that support mitochondrial efficiency and reduce oxidative stress, which in turn may stabilize metabolic signaling and inflammatory tone. Thus, the microbiome can function not only as a mediator of risk but also as an amplifier of redox resilience within the broader preventive framework.

## 3. Lifestyle and Natural Dietary Antioxidants as the Primary “Lever”

### 3.1. Plant-Predominant Dietary Patterns and Multi-Disease Prevention

Lifestyle-oriented preventive medicine emphasizes that dietary patterns can reduce the risk for multiple chronic diseases simultaneously. Whole-food, plant-forward diets are typically rich in antioxidant and anti-inflammatory compounds, higher in fiber, and lower in pro-oxidant constituents such as excess saturated fat and heme iron. These characteristics collectively improve metabolic markers, endothelial function, and basal inflammatory tone [5,32,33]. For example, long-term adherence to a Mediterranean or similar plant-predominant diet is associated with lower incidence of cardiovascular disease, type 2 diabetes, and neurodegenerative disease [5,32]. At a mechanistic level, such diets provide abundant phytochemicals (such as flavonoids/phenolic acids, carotenoids, and organosulfur compounds) that modulate redox signaling, while also promoting a healthier gut microbiome (in part due to high fiber intake). A recent clinical analysis from the CARDIA study illustrates the organ-level benefits: higher intake of fiber-rich, unrefined plant foods in young adults was associated with better left ventricular structure and function decades later in midlife [41]. In populations consuming primarily whole-plant-based diets (as observed in certain Blue Zone regions or traditional societies), rates of coronary artery disease, colon cancer, and other major causes of morbidity and mortality (e.g., stroke and colorectal cancer) remain strikingly low. Such epidemiological and interventional evidence underlines the potential of diet as a multi-disease prevention lever. By emphasizing vegetables, fruits, whole grains, legumes, nuts, and seeds—and minimizing ultra-processed foods—individuals can simultaneously address numerous upstream risk factors: oxidative stress, inflammation, dyslipidemia, hypertension, insulin resistance, and related cardiometabolic risk factors [5,32,33]. In sum, a plant-predominant diet is a cornerstone for deploying natural antioxidants in a truly translational way to combat chronic diseases.

From a quantitative perspective, population-level dietary patterns associated with the lowest cardiometabolic risk are typically characterized by high intake of minimally processed plant foods and dietary fiber (minimum guideline targets often ≥25–35 g/day in adults), regular consumption of fruits and vegetables (≥5 servings/day), legumes and whole grains, and limited intake of ultra-processed foods and refined carbohydrates [4,5,12]. While many contemporary dietary guidelines recommend fiber intakes in the range of 25–35 g/day, anthropological and observational data from traditional agrarian and hunter–gatherer populations suggest substantially higher habitual intakes, frequently estimated at 70–100 g/day or more. Such intake levels have been associated with lower systemic inflammation, favorable gut microbial metabolite profiles, and minimal prevalence of atherosclerotic disease in non-industrialized settings [42]. These observations do not imply that high fiber intake alone is causative, but they raise the possibility that current minimum recommendations reflect feasibility in Western dietary contexts rather than physiologic ancestral exposure levels.

Rather than focusing on isolated “antioxidant doses”, these patterns reflect cumulative exposure to diverse redox-active phytochemicals (e.g., polyphenols, carotenoids, organosulfur compounds) within a whole-food matrix. Importantly, this framework emphasizes dietary patterns and nutrient density rather than high-dose supplementation, as physiological redox balance appears more consistently supported by sustained dietary intake than by pharmacological antioxidant dosing in otherwise replete individuals.

### 3.2. Natural Antioxidant Components of Whole Foods

Whole foods contain a synergistic spectrum of natural antioxidants, including vitamins (e.g., vitamin C, vitamin E), polyphenols (flavonoids, stilbenes like resveratrol), carotenoids (beta-carotene, lycopene), and organosulfur compounds (like allicin from garlic, sulforaphane from broccoli). Notably, these compounds often do not act solely as direct free-radical scavengers; rather, many exert effects via hormesis—mild, adaptive stress that upregulates endogenous defenses—and by modulating redox-responsive signaling pathways. For instance, polyphenols can activate the Nrf2, the master regulator of cytoprotective genes, leading to increased expression of antioxidant enzymes (superoxide dismutase (SOD), glutathione peroxidase, heme oxygenase (HO)-1, etc.) [43]. Additional food-derived antioxidants and redox-active constituents—including cocoa flavanols, anthocyanin-rich berries, and nitrate-rich leafy greens/beetroot—have been associated with improved endothelial nitric oxide bioavailability and vascular function in both experimental and clinical contexts. Dietary flavanols such as those found in cocoa have been shown to enhance endothelial function and nitric oxide availability through modulation of oxidative stress pathways (e.g., flow-mediated dilation and blood pressure improvement) in human and animal studies [44]. Anthocyanin-rich berry consumption has been linked to favorable effects on endothelial health and cardiovascular risk markers in preclinical and human studies [45]. Nitrate-rich foods such as beetroot juice reliably increase systemic nitrate/nitrite and nitric oxide bioavailability, improving vascular function and endothelial responsiveness in randomized interventions [46,47].

It is the combination of these actions—direct ROS neutralization, gene expression changes, and anti-inflammatory signaling—that likely explains the broad benefits of diets rich in varied antioxidants. Importantly, whole-food matrices may improve the bioavailability and distribution of these phytochemicals compared to isolated supplements, and they avoid the hazard of high-dose single antioxidants potentially disrupting redox balance. Thus, nutrition delivers antioxidants in a form and dose attuned to our biology’s needs, helping maintain redox homeostasis across multiple organ systems [31]. Ongoing research continues to identify new bioactive compounds (e.g., taurine as recently highlighted for longevity) and to elucidate how diet-driven shifts in the redox proteome translate into disease prevention.

Importantly, fiber-rich whole-food dietary patterns influence redox balance not only through phytochemicals but also via microbial fermentation products such as short-chain fatty acids, which have been shown to modulate mitochondrial function and inflammatory tone [38].

### 3.3. “Genes Are Guns…”: Lifestyle as the Trigger (and the Preventive Safety)

The adage “genes load the gun, but lifestyle pulls the trigger” captures the essence of gene–environment interaction in chronic disease. Genetic predispositions often remain clinically silent unless “triggered” by adverse lifestyle factors—and conversely, healthy lifestyle choices can be a safety catch that keeps genetic risk from translating into disease. Modern research in epigenetics and gene expression confirms this thesis: diet, physical activity, stress, and toxin exposure can modulate gene expression through DNA methylation, histone modification, and microRNA, thereby influencing whether disease-related genes are up- or down-regulated. For example, studies integrating genetic risk with lifestyle factors show that adherence to a healthy lifestyle is associated with substantially lower coronary disease risk even among individuals with high polygenic risk [48]. On the flip side, individuals with “good genes” can still develop conditions like type 2 diabetes or atherosclerosis if they lead a sedentary life with a poor diet. Lifestyle is thus the crucial interface between genetic endowment and health outcomes. Maintaining a favorable lifestyle—plant-rich diet, regular exercise, no smoking, moderate stress—creates an internal environment that minimizes chronic redox-inflammatory activation, which is the common soil of so many diseases. This provides a conceptual basis for simultaneous prevention: by pulling a small set of upstream lifestyles “levers,” we can favorably influence a wide array of downstream disease processes. The possibility of influencing multiple diseases through coordinated lifestyle interventions is increasingly supported by clinical and epidemiological evidence, although effect sizes and durability vary across populations and contexts [5,32]. In summary, while we cannot change our genes, we can profoundly influence how and whether our genes manifest as disease—and that agency lies largely in lifestyle choices.

## 4. Targeted Nutraceuticals and Supplements (with Cardiovascular Exemplar)

### 4.1. Cardiovascular Exemplar: Reperfusion-Induced Injury (RII) in the Heart

To anchor the redox framework in a clinically urgent cardiovascular setting, we include a focused section on myocardial ischemia/reperfusion injury and the phenomenon of reperfusion-induced injury (RII). In this scenario, sudden restoration of blood flow (reperfusion) to an ischemic heart can itself paradoxically cause additional damage. Here, ROS generation and downstream inflammatory and electrophysiological consequences are central, and antioxidant-oriented strategies have been extensively studied as potential mitigators of myocardial ischemia/reperfusion injury [49,50].

RII occurs not only in the heart [51,52,53,54,55,56] but also in every organ, in which the supply of oxygen and nutrients is indispensable for survival and for the maintenance of vital physiological functions. Thus, in the central nervous system [57,58,59,60], the kidney [61,62,63,64], the lung [65,66,67], the liver [68,69,70,71], and the gastrointestinal system [72,73,74], the process of ischemia/reperfusion can cause significant tissue damage, thereby impairing fundamental physiological functions of these organs. The evidence is that RII triggers numerous pathological alterations and signal transduction pathways in various tissues and organs. However, the relative contribution of the underlying pathological mechanisms differs among tissues; therefore, the pharmacological management of ischemia/reperfusion-induced injury is essentially tissue- or organ-specific.

In fact, it can be clearly established that the formation of reactive oxygen species (free radicals) [53,75,76] and intracellular Ca^2+^ accumulation play a fundamental role in the development of “necroapoptophagic” cell death processes [15], including necrosis, apoptosis, and autophagy.

RII encompasses various forms of cell death and tissue damage that occur upon reperfusion of previously ischemic myocardium, as opposed to the injury directly caused by the preceding ischemic period. It is well known that ischemic tissues cannot recover unless perfusion (reflow) is reestablished. However, reperfusion can actually extend the irreversible damage initiated by ischemia—the returning blood flow triggers oxidative bursts and calcium overload that exacerbate cell death—even as it is necessary to salvage viable tissue [49]. Thus, while reperfusion is essential for rescuing ischemic myocardium, it also paradoxically contributes to myocardial dysfunction, arrhythmias (e.g., ventricular fibrillation, a cause of sudden cardiac death), and the development of heart failure in the aftermath of infarction. In recent decades, RII has been found to involve not only oxidative stress and calcium imbalance but also expanded roles for immune activation, mitochondrial dysfunction, necrosis and apoptosis, ferroptosis, and various gene-expression changes during reperfusion. The extent and mechanisms of RII can vary based on the duration of ischemia and the conditions of reperfusion [49,50].

Key mechanisms of RII include a massive burst of ROS generation at the moment of reperfusion (as oxygen re-enters ischemic tissue and mitochondria), which in turn triggers opening of the mitochondrial permeability transition pore, cytokine release, and infiltration of neutrophils. This oxidative and inflammatory cascade leads to lipid peroxidation, protein oxidation, and DNA damage in cardiomyocytes. RII is a major contributor to reperfusion-induced arrhythmias (such as ventricular fibrillation) and contractile dysfunction. From the 1980s onward, extensive experimental and clinical investigations have established that myocardial ischemia followed by reperfusion produces rapid biochemical and electrophysiological changes leading to cell death and arrhythmogenesis. A consensus emerged that ROS generation is a fundamental trigger in RII pathogenesis. Excess ROS during reperfusion directly injure cardiomyocytes and serve as second messengers activating maladaptive signaling—for instance, oxidized lipids and proteins can provoke calcium overload and mitochondrial injury [52,77,78,79].

Multiple lines of evidence link RII to specific molecular pathways: activation of NF-κB and inflammasomes during reperfusion amplifies inflammation; opening of cardiomyocyte sarcolemmal K_ATP_ channels and mitochondrial K_ATP_ channels can occur; and the renin–angiotensin system and sympathetic surge at reperfusion further modulate these processes. Arrhythmias in early reperfusion are partly precipitated by ROS-induced modifications of ion channels and gap junctions. For example, changes in potassium and calcium channel function (due to oxidation of channel proteins or altered kinase signaling) create dispersion of repolarization, setting the stage for ventricular tachycardia/fibrillation [15,80,81,82,83,84,85,86,87,88,89]. Accordingly, this section will highlight some critical signal transduction mechanisms in arrhythmogenesis during ischemia/reperfusion and assess the anti-arrhythmic potential of various phytochemicals and drugs with antioxidant properties.

Research into RII has identified a complex interplay of cell death modalities—necrosis from bioenergetic failure, apoptosis from programmed pathways, and autophagy (which can be either protective or contributory to cell death). Recently, ferroptosis (iron-catalyzed lipid peroxidation-driven cell death) has also been implicated in reperfused myocardium [80,81,82,83,84]. The relative contribution of each mechanism can depend on ischemia duration and specific context. Importantly, numerous pharmacological interventions targeting these pathways have been tested. Antioxidant strategies have included direct free-radical scavengers (e.g., SOD mimetics), iron chelators to prevent hydroxyl radical formation, and inhibitors of oxidative enzymes like xanthine oxidase. Anti-inflammatory approaches and agents modulating stress-response pathways (including conditioning-based strategies) have also been explored in the reperfused heart [50].

Classic studies (e.g., by Jennings et al. in the 1980s) demonstrated that very early reperfusion is necessary to salvage myocardium, but also noted ultrastructural damage arising after reperfusion, attributable to calcium oscillations and swelling in mitochondria [77]. Later, it was shown that applying interventions now of reperfusion—such as antioxidants, calcium modulators, or ischemic post-conditioning (brief intermittent reocclusion cycles)—could reduce infarct size, supporting the causative role of RII processes. More recent work has expanded our understanding: for instance, Oerlemans et al. (2013) highlighted the role of the innate immune response in RII [50], and Koshinuma et al. (2014) discussed anesthetic-induced conditioning against RII [90]. Haines et al. (2013) likewise emphasized oxidative stress management in multicellular contexts [15].

Given the breadth of RII mechanisms, an array of phytochemicals and nutraceuticals have been tested for cardioprotective effects in this setting. Some notable examples include: resveratrol (a polyphenol from grapes), which activates endothelial nitric oxide synthase and can reduce infarct size [21,91]; curcumin (from turmeric), which has antioxidant and anti-inflammatory effects and was shown to attenuate post-infarction remodeling in animal models [92,93]; and quercetin (a flavonoid), which can inhibit lipid peroxidation and protect mitochondrial function in ischemia/reperfusion models [94,95]. Many of these compounds also activate Nrf2, augmenting endogenous antioxidant defenses in the heart [43,91,93]. There is evidence that chronic intake of polyphenol-rich foods such as green tea and berries can enhance antioxidant and phase 2 detoxifying defenses relevant to ischemia/reperfusion protection [96,97]. However, translation to clinical practice has been challenging—large clinical trials of antioxidant vitamins (like E and C) in acute coronary syndromes did not yield clear benefits [31]. It appears that timing, bioavailability, and target specificity are critical: an intervention must be at the right place and time to intercept the burst of ROS at reperfusion or to modulate the key signaling events (such as mitochondrial permeability transition) to be effective.

In summary, myocardial reperfusion injury exemplifies how oxidative stress lies at the heart of acute and chronic pathology in cardiovascular disease. It also provides a testing ground for antioxidant therapies—both natural and synthetic. Insights gained here, such as the importance of bolstering endogenous defenses before injury occurs, carry over to chronic disease prevention. RII continues to be an area of active investigation, with ongoing studies into combined therapies (e.g., antioxidant + anti-apoptotic agents, or conditioning plus drugs) and novel delivery methods (such as nanoparticle antioxidants) to mitigate this double-edged sword of reperfusion.

Importantly, ischemia/reperfusion injury is not heart-specific; similar ROS-driven cascades contribute to tissue loss and inflammatory amplification in cerebral ischemia (stroke) and in renal ischemia/reperfusion contexts, reinforcing the generalizability of redox-targeted strategies beyond cardiology.

### 4.2. Targeted Nutraceuticals: Scope and Positioning

Beyond whole-diet patterns, nutraceuticals and concentrated supplements are often used to target specific redox and inflammatory pathways in individuals at high risk or with existing disease. Within a “simultaneous prevention” framework, such supplements are best positioned as adjuncts—potentially useful when they (i) complement a foundation of a healthy diet, (ii) address a measurable deficiency or abnormal biomarker, or (iii) target a specific clinical problem (for example, mitochondrial dysfunction, endothelial dysfunction, or post-ischemic oxidative injury) [98]. In other words, nutraceuticals should fine-tune the system rather than replace broad dietary benefits. Common antioxidant nutraceuticals include coenzyme Q10 (coQ10), to support mitochondrial electron transport and act as a lipid-phase antioxidant [99], omega-3 fatty acids, to resolve inflammation and reduce oxidative lipid mediators, lipoic acid, a thiol antioxidant that also regenerates glutathione and reduces myocardial ischemia–reperfusion injury [100,101], and plant-derived polyphenol extracts, such as grape seed extract or green tea catechins, which enhance myocardial antioxidant defenses [96].

While numerous nutraceuticals have demonstrated antioxidant activity in vitro, translational efficacy depends heavily on bioavailability, formulation, and clinical context. For example, coQ10 has shown benefit in chronic heart failure in the Q-SYMBIO randomized trial [99], whereas polyphenols such as curcumin and resveratrol often require enhanced formulations to overcome limited oral bioavailability. Moreover, dose–response relationships in animal I/R models cannot be directly extrapolated to human preventive contexts. These considerations highlight that nutraceutical interventions function best as adjuncts within dietary patterns rather than isolated high-dose pharmacologic agents.

Some vitamins and minerals are used in a targeted way, particularly when deficiency or suboptimal status is documented; examples include vitamin D (immune and inflammatory modulation), magnesium (metabolic and vascular function), and selenium/zinc/copper as cofactors supporting endogenous antioxidant enzymes [98]. Detailed representative dosing and duration ranges for selected interventions are summarized in Table 2.

Evidence for nutraceutical efficacy is mixed—in part because large-scale trials have sometimes failed when testing one compound in unselected populations [31]. This underscores that supplements are context-dependent tools. For example, in selenium-deficient individuals, selenium supplementation can restore selenoprotein activity and reduce oxidative stress, whereas excessive selenium intake may be harmful, underscoring the importance of baseline status [103,107,108]. By contrast, giving selenium to already replete individuals may have little benefit or even confer some risk [108]. Similarly, high-dose antioxidant cocktails given without understanding the patient’s baseline redox status might blunt beneficial reactive oxygen signaling (such as the oxidative burst required for insulin sensitivity and exercise adaptations). Therefore, an integrative strategy is recommended: use nutraceuticals selectively—for example, in older patients with known mitochondrial impairment, where coQ10 has shown benefits as adjunctive therapy in chronic heart failure [99], or in those with inflammation-driven conditions where polyphenols such as curcumin or quercetin can inhibit NF-κB and reduce oxidative-inflammatory damage [93,94]. The following sections (Section 5 and Section 6) bridge between population-level lifestyle prevention and these more targeted approaches, including applications in rare “stress-test” diseases and future gene-based redox modulation.

## 5. Natural Antioxidants in Orphan Diseases as a “Stress Test” of Redox-Based Strategies

Rare hereditary diseases—such as familial neurodegenerative syndromes and mitochondrial encephalopathies—are often characterized by extreme, chronic oxidative stress and free radical overload. In particular, a number of LSDs (e.g., Tay–Sachs disease (TSD) and Niemann–Pick disease (NPD)) are associated with excessive ROS production that contributes to the progression of neurodegeneration [109]. In these conditions, mutations leading to enzyme deficiencies cause the accumulation of toxic substrates in cells, which is accompanied by activation of inflammatory pathways and oxidative damage to tissues [24]. Studies in disease models support this pathological sequence: for example, in a TSD mouse model, redox homeostasis imbalance was closely linked to neuron death, suggesting that targeted reduction of ROS levels might mitigate the course of neurodegeneration [109]. Similarly, in Niemann–Pick disease type C (NPC), a rare lysosomal lipid storage disorder, intracellular cholesterol accumulation is associated with mitochondrial and lysosomal dysfunction as well as pronounced oxidative stress. Limited studies report that antioxidant-based approaches in NPC models can reduce ROS and cellular damage, indicating that lowering oxidative injury may be an important component of therapy for this disorder [22].

In this context, there is growing interest in the use of natural antioxidants—compounds of natural origin capable of neutralizing free radicals or boosting endogenous antioxidant defenses—as adjunctive therapeutic strategies for rare diseases characterized by chronic oxidative stress. Such compounds primarily include plant-derived polyphenols (e.g., curcumin from turmeric, resveratrol from grapes, quercetin from fruits and vegetables), among others. These molecules exhibit pleiotropic biological effects, combining direct free radical–scavenging activity with modulation of cellular signaling pathways. For example, polyphenols can activate Nrf2, thereby inducing expression of cytoprotective genes encoding antioxidant enzymes (SOD, glutathione peroxidase, HO-1, etc.) [43]. They may also inhibit pro-inflammatory mediators (like NF-κB) and improve defective autophagy or lysosomal function that often accompany these diseases.

Studies using models of certain rare diseases have indeed demonstrated that naturally derived compounds can attenuate oxidative tissue damage. For instance, sulforaphane—a potent Nrf2-activating antioxidant found in cruciferous vegetables—was shown to enhance antioxidant responses and reduce intracellular ROS levels in fibroblasts derived from NPC models [22]. Resveratrol and related stilbene compounds (such as pterostilbene), as well as flavonoids like catechin, have been proposed as therapeutic agents for some LSDs. In vitro data indicate their ability to reduce pathological substrate accumulation and confer cellular protection in models of mucopolysaccharidosis type VII [110] and Gaucher disease [111]. In an NPC mouse study, resveratrol alone had modest effects on lifespan extension; however, when curcumin (another polyphenol) was combined with a therapy that reduces sphingolipid synthesis, more pronounced neuroprotective effects and delayed disease progression were observed [112]. Taken together, natural antioxidants are viewed as promising adjuncts in the treatment of many orphan diseases whose pathogenesis involves chronic oxidative stress.

Mitochondrial encephalopathies—rare syndromes caused by mitochondrial respiratory chain dysfunction (e.g., MELAS and other disorders affecting oxidative phosphorylation)—have attracted particular attention vis-à-vis antioxidant therapy. These diseases feature impaired ATP production and excessive ROS generation due to dysfunctional mitochondria, leading to damage in neurons and muscle cells. Traditionally, a cocktail of empiric antioxidants and cofactors (vitamins E and C, coQ10, riboflavin, etc.) has been used in managing patients with mitochondrial disorders, aiming to mitigate oxidative stress. While the clinical evidence base is limited, some systematic studies in model organisms support this rationale [113]. For example, a recent high-throughput screening in zebrafish and *Caenorhabditis elegans* models of respiratory chain defects found that out of seven tested antioxidants, N-acetylcysteine (NAC) (a glutathione precursor) and vitamin E produced the most pronounced extension of lifespan and protection of neurons from cell death. CoQ10 (including mitochondria-targeted formulations) partially improved energy metabolism in these models, though it did not fully restore organismal viability. Moreover, in patient-derived cell cultures with complex I deficiencies, supplementation with NAC or vitamin E increased cell survival by reducing intracellular ROS levels [105]. These preclinical findings align with anecdotal clinical observations that some mitochondrial disease patients report improved fatigue or exercise tolerance on coQ10 or antioxidant vitamins, although robust trial data are scarce.

Clinical experience with natural antioxidants in orphan diseases remains mostly limited to case reports and small series, yet the results are often encouraging. For example, a recent case report of adult TSD demonstrated that adding curcumin to the patient’s comprehensive therapy was associated with reduced levels of proinflammatory cytokines and some improvement in overall condition. In this report, curcumin was administered alongside an umbilical cord blood transplant, and afterward, a shift in the serum cytokine profile was observed—specifically, several inflammatory factors decreased—while the primary toxic substrate (GM2 ganglioside) remained largely unchanged [23]. This pattern suggests that natural antioxidants may function primarily as adjunctive modulators of inflammation and oxidative injury, rather than as stand-alone cures, in monogenic storage disorders. Similarly, isolated cases in other conditions (like MELAS or Friedreich’s ataxia) have noted improvements in biomarkers or symptoms with antioxidant supplementation, though controlled studies are needed.

In summary, rare inherited diseases serve as a valuable “stress test” for redox-based strategies: if boosting antioxidant defenses or quelling oxidative damage can yield even slight improvements in these extreme settings, it reinforces the principle that oxidative stress is a common denominator of pathology. Natural antioxidants in orphan diseases are considered supportive treatments to attenuate chronic inflammation and oxidative damage, potentially improving patients’ quality of life. At present, such interventions should be viewed only as adjuncts—further in vitro work, animal studies, and clinical trials are required to confirm the efficacy and safety of natural antioxidants in the context of specific rare diseases. The lessons learned, however, can inform approaches to more common diseases that share mechanistic threads with these orphan disorders.

### 5.1. Candidate Natural Antioxidants and Mechanisms

Candidate compounds of interest for these purposes include the polyphenols and related phytochemicals mentioned above (curcumin, resveratrol, quercetin, catechins, sulforaphane, etc.), as well as certain endogenous antioxidants (like glutathione precursors or cofactor vitamins) that can be given exogenously. These agents exert multi-target effects—they can directly scavenge ROS, activate Nrf2-dependent antioxidant gene programs, attenuate pro-inflammatory signaling cascades, and even modulate impaired autophagy or lysosomal clearance. A key translational issue for applying antioxidants in diseases with, for example, central nervous system (CNS) involvement is bioavailability and tissue penetration. Many phytochemicals have limited ability to cross the blood–brain barrier or achieve high concentrations in affected organs. This makes formulation and delivery strategies critical: researchers are exploring nano-formulations, prodrugs, or synergistic combinations to enhance the delivery of antioxidants to target sites [31]. For instance, liposomal curcumin or brain-penetrant resveratrol analogs are being investigated to treat neurological aspects of LSDs. Ensuring that antioxidants reach the sites of oxidative stress in sufficient quantity (without causing off-target effects) is essential for translating their promise into meaningful clinical outcomes [31].

### 5.2. Preclinical Evidence (Cell and Animal Models)

Across models of lysosomal and mitochondrial disorders, natural antioxidants have been reported to reduce markers of oxidative damage, improve mitochondrial parameters, and modulate inflammatory pathways. For example, as noted, sulforaphane treatment enhanced antioxidant responses and lowered intracellular ROS in NPC fibroblasts [22]. Resveratrol and other stilbenes have shown evidence of improving cellular phenotypes in certain LSD models, such as mucopolysaccharidosis type VII in Drosophila [110,114]. Curcumin has yielded mixed outcomes: in some animal models used as a single therapy, it showed only modest benefits, but when combined with other treatments—for instance, in a triple combination with miglustat and ibuprofen in NPC1 mice—it produced more notable neuroprotective effects and significantly prolonged survival [112,115]. These examples illustrate a general pattern: antioxidants alone may confer partial protection, but their effects can be potentiated when used as part of a combination strategy, such as alongside substrate reduction therapy, chaperone therapy, or gene therapy.

Notably, the NAC and vitamin E findings in the *C. elegans* and zebrafish models of mitochondrial disease stand out because they were identified through unbiased screening. NAC’s ability to boost glutathione and vitamin E’s lipid peroxidation chain-breaking activity emerged as the top intervention to extend lifespan in those models. These results have since influenced clinical trial designs in mitochondrial disease—for example, trials of NAC in mitochondrial myopathy. The replication of antioxidant benefits in both worm and fish models, and at the cellular level in patient fibroblasts, strengthens the biological plausibility that targeting oxidative stress can modify disease trajectory in mitochondrial disorders [105].

In lysosomal diseases, where neurodegeneration is often rapid and severe, animal model evidence for antioxidants is more limited (due to the lack of long-living models in some cases). However, catalase gene delivery has been tested in CNS inflammatory models, where viral overexpression suppressed oxidative injury and improved pathology [116]. These experiments reinforce that oxidative damage is not merely a byproduct but a contributor to pathology, as reducing ROS tended to preserve neurons or prolong survival modestly.

### 5.3. Clinical Evidence in Humans

Human evidence in orphan diseases is limited and often consists of case reports or small open-label studies. As an illustrative example (mentioned above), Shaimardanova et al. (2021) [23] reported on curcumin use in an adult patient with TSD as part of a multi-modal therapeutic approach. In that case, curcumin administration (alongside hematopoietic stem cell transplantation) was associated with a shift in the patient’s serum cytokine profile toward a less inflammatory state and subjective clinical improvement, despite little change in stored ganglioside levels. This suggests curcumin’s primary effect was to dampen the harmful inflammatory milieu rather than clear the substrate [23]. Such reports align with the notion that natural antioxidants mainly function as adjunct modulators—reducing damage and inflammation—rather than correcting the root enzymatic defect in monogenic diseases.

CoQ10 (including idebenone-related approaches) has been evaluated in Friedreich’s ataxia, with mixed evidence across studies and reviews [117,118]. In MELAS syndrome, antioxidants like vitamin E, l-carnitine, and alpha-lipoic acid are often given empirically; some patients report better stamina or milder stroke-like episodes, but controlled data are lacking [25]. An encouraging anecdote comes from experimental and clinical work showing that selenium supplementation can increase glutathione peroxidase activity and improve muscle or mitochondrial function, suggesting that optimizing selenium status may help correct antioxidant enzyme deficits [104,119]. However, these findings need rigorous verification in well-designed clinical trials.

The overall pattern is that antioxidants by themselves rarely produce dramatic clinical reversal in genetic diseases, but they can tilt the balance in favor of better outcomes or slow deterioration. Patients and clinicians consider them as part of a “stack” of interventions. It will be important for future trials to formally test combinations (for instance, antioxidant + anti-inflammatory + enzyme replacement) to see whether there are synergistic benefits. At the very least, ensuring patients are replete in key dietary antioxidants and cofactors (including relevant trace elements such as selenium) is a low-risk measure to avoid compounding oxidative stress [103,120].

It is important to emphasize that, despite encouraging mechanistic and preclinical findings, controlled human trials in rare redox-imbalance disorders remain limited. Most available evidence derives from small case series, animal models, or mechanistic studies. Therefore, antioxidant interventions in orphan diseases should currently be considered supportive rather than disease-modifying therapies, pending rigorous prospective validation.

### 5.4. Translational Lessons from Orphan Diseases to Common Diseases

Orphan diseases highlight a key principle relevant to broad chronic disease prevention: when oxidative stress and inflammation are persistent drivers of tissue damage and functional decline, then upstream redox modulation—whether via diet, nutraceuticals, or future gene-based approaches—can reduce the overall “system load” even if the primary genetic cause remains [121,122]. In other words, taming oxidative stress can buy time and preserve function. This supports a unified redox framework across rare and common conditions. The same pathways that wreak havoc in rare metabolic disorders (mitochondrial ROS, defective autophagy, uncontrolled inflammation) are present at lower amplitudes in common age-related diseases [123,124]. Interventions that show promise in extreme models (like LSDs or mitochondrial syndromes) may be translated or scaled to typical chronic diseases of aging [105].

For example, insights from NPC research on how lipid accumulation can trigger ROS and inflammation have informed a broader understanding of atherosclerosis and neurodegenerative processes, where related mechanisms may occur despite different primary causes [22,31]. The concept of boosting endogenous antioxidant defenses—for instance, via Nrf2-linked strategies—could one day be applied to age-associated neurodegenerative disorders and cardiomyopathy [121,125]. Moreover, these rare diseases underscore the importance of combination therapy: no single antioxidant or drug is likely sufficient, but integrating multiple approaches (diet, antioxidants, anti-inflammatories, gene therapy, etc.) might achieve meaningful results, as suggested by triple-combination regimens in NPC1 mice (miglustat + curcumin + ibuprofen) [112].

In summary, rare disorders serve as “stress tests” that validate oxidative stress as a unifying target. They also caution that interventions need to be potent and targeted enough to matter. As we move to more common diseases, the lessons learned—especially the need for early and multi-modal intervention—reinforce the rationale for lifestyle medicine as the first line of defense, supplemented by precise adjuncts. Aging itself can be viewed as a cumulative, slowly progressive “orphan disease” of oxidative stress, mitochondrial dysfunction, and macromolecular damage [122]. Thus, the strategies explored in extreme orphan diseases may, with appropriate adaptation, help craft preventive and therapeutic approaches for the broader population [121].

## 6. Gene Therapy as an Antioxidant Strategy: Engineered Redox Modulation

The following section should be interpreted as a forward-looking translational perspective rather than a near-term clinical recommendation. Gene-based reinforcement of endogenous antioxidant defenses is discussed here as a conceptual extension of dietary redox modulation, representing a potential future adjunct in severe or refractory phenotypes where lifestyle and nutraceutical strategies alone may be insufficient.

Lifestyle and dietary antioxidants represent the most scalable and immediate route to redox modulation, but they are limited by bioavailability, tissue targeting, and long-term adherence. An emerging complementary concept is gene-based strengthening of endogenous antioxidant systems—a strategy that can be viewed as “engineered antioxidant therapy” [28,106]. This approach seeks to enhance the body’s own protective mechanisms by delivering genes that combat oxidative stress. Conceptually, this could be relevant both to common cardiovascular/metabolic diseases and to selected orphan disorders, particularly where oxidative injury is a persistent, tissue-specific driver of pathology [27,126].

### 6.1. Targets: Antioxidant Enzymes and Redox Regulators

Gene delivery approaches can aim to increase the expression of key antioxidant enzymes: for example, the enzymes that directly neutralize ROS such as superoxide dismutases (SOD1 in the cytosol and SOD2 in mitochondria), catalase (which decomposes hydrogen peroxide), and glutathione peroxidases (GPX1 and others). Experimental gene therapy studies have shown that delivering SOD1 or catalase genes can reduce oxidative vascular damage and even slow the formation of atherosclerotic plaques in predisposed mice [127,128,129]. Similarly, overexpression of glutathione peroxidase or HO-1 via gene vectors has demonstrated anti-inflammatory and vasoprotective effects in models of chronic vascular inflammation [129]. These successes underscore that directly augmenting antioxidant enzymes in target tissues (like blood vessels) can suppress atherogenesis and injury [129].

Alternatively, gene therapy can target upstream regulatory factors that orchestrate broad antioxidant and cytoprotective responses. Instead of (or in addition to) individual enzymes, one can introduce genes encoding transcriptional activators such as Nrf2—the master regulator of cellular antioxidant responses—or its positive regulators. By enhancing Nrf2 activity in endothelial and smooth muscle cells, for instance, researchers have increased the expression of a spectrum of antioxidant genes and observed reductions in oxidative damage markers [129]. Another target is peroxisome proliferator-activated receptor gamma coactivator 1-alpha (PGC-1α), a co-activator that promotes mitochondrial biogenesis and antioxidative metabolism; boosting PGC-1α could improve mitochondrial resilience. Additionally, delivering genes encoding inhibitors of pro-oxidant pathways (for example, a dominant-negative IκB to inhibit NF-κB, or extracellular superoxide dismutase to scavenge ROS in the extracellular space) has been explored [129]. All these strategies aim at tipping the balance within cells toward a state of enhanced oxidative defense and reduced inflammatory signaling.

In principle, boosting endogenous redox buffering capacity via gene therapy could reduce oxidative damage, dampen chronic inflammatory signaling, and preserve mitochondrial function in tissues burdened by stress. This approach has been tested most extensively in vascular contexts: for example, Van-Assche et al. (2011) demonstrated that gene delivery of antioxidant enzymes or regulators significantly suppressed atherogenesis in animal models [129]. The appeal of gene therapy here is that it could provide a long-lasting intervention—a one-time delivery could induce sustained high levels of an antioxidant protein in a target organ, something difficult to achieve with drugs or supplements.

### 6.2. Relevance to Cardiovascular Syndromes and Chronic Disease

Oxidative stress is a major contributor to cardiovascular diseases. It plays a role in endothelial dysfunction (by reducing nitric oxide and damaging endothelial cells), in LDL oxidation (triggering plaque formation), in activating immune cells within atherosclerotic lesions, and in adverse remodeling processes implicated in hypertension, myocardial infarction (MI), and heart failure progression [129]. Given this, a gene therapy that bolsters antioxidant defenses in the heart and blood vessels could conceptually complement lifestyle prevention by providing durable, tissue-level protection. For example, delivering an SOD2 gene specifically to cardiac myocytes or vascular endothelium might help prevent the oxidative damage that accumulates with hypertension or diabetes. Indeed, studies have shown that gene transfer of antioxidant enzymes inhibits the progression of atherosclerosis in mice [127,128,129]. Similarly, in models of ischemia/reperfusion (like discussed in Section 4.1), viral vectors encoding catalase or SOD have reduced infarct sizes and arrhythmias by quenching ROS during reperfusion.

Heart failure is another target—chronic heart failure is characterized by elevated myocardial oxidative stress and a failing antioxidant system. Experimental therapies increasing SOD or catalase in failing hearts improved contractile function in some studies, supporting the role of ROS in driving pump failure. The inflammation–oxidative nexus is also critical in conditions like chronic kidney disease and diabetes complications; gene therapy aimed at Nrf2 activation has shown promise in improving diabetic wound healing and nephropathy in animal models [130,131,132].

An advantage of gene therapy is tissue targeting. For instance, an adeno-associated virus (AAV) can be engineered to target myocardium or endothelium, delivering an antioxidant gene directly where it is needed while sparing other tissues, thereby potentially minimizing side effects [27,133]. By contrast, systemic antioxidants (like oral supplements) distribute throughout the body and may not reach adequate concentrations in the target tissue. Redox-oriented gene therapy, therefore, could act as a precise strike—for example, enhancing antioxidant capacity in cardiac muscle to reduce ischemia/reperfusion injury [27,106,134] or in the CNS to suppress oxidative-inflammatory damage in experimental optic neuritis [116].

In summary, this approach does not replace a healthy lifestyle but could complement it by reinforcing the body’s defenses in situations where diet and exercise alone might not fully suffice—particularly in advanced age-related pathology or in rare diseases with extreme oxidative stress. For example, an elderly patient with aggressive atherosclerosis despite a good diet might one day receive an endothelial-targeted Nrf2 or extracellular SOD gene therapy to stabilize plaques and reduce inflammation [27,134]. Such scenarios remain hypothetical at present, but proof-of-concept studies in animals are steadily building the case [106,126].

### 6.3. Safety and Translational Considerations

Despite its promise, practical translation of gene-based antioxidant strategies faces several challenges. Safety is paramount: delivering genes into human cells must be done with vectors (such as viruses, plasmids, or newer mRNA approaches) that minimize adverse effects. There are concerns about dose control—excessive expression of even a “good” gene could have off-target effects or disrupt cell function. For instance, overcorrection of redox balance could, in theory, dampen physiological ROS signaling that cells require for normal processes (ROS are also important signaling molecules in immune defense and adaptation to stress). Thus, the goal is restoration of homeostasis rather than pushing antioxidant levels to unnatural highs. To address this, researchers are exploring tissue-specific promoters and other regulatory elements to better control transgene expression and improve safety [28].

Tissue targeting and delivery method are also critical. Systemic delivery may be required for multi-organ conditions (with associated risks of immune reaction to vectors), whereas local delivery can be used for accessible sites (for example, intracoronary injection for the heart) [133,135]. The immune response to vectors—especially viral vectors such as AAV—is a known issue: prior exposure can lead to neutralizing antibodies, and strong immune reactions can cause inflammation or loss of therapeutic expression. Accordingly, newer vectors (AAV capsid variants, non-viral nanoparticles) and transient expression systems (e.g., modified mRNA) are being explored for improved safety profiles [28].

Durability of expression is another factor. While an ideal gene therapy might permanently install an antioxidant defense, many current vectors eventually lose expression (due to cell division or epigenetic silencing of vector genomes). Repeated dosing might be needed, which again raises immunogenicity concerns [28].

Finally, rigorous preclinical testing is necessary to ensure that upregulating an antioxidant pathway is not accompanied by unintended consequences. For example, chronic Nrf2 activation could potentially interfere with normal immune surveillance or hormetic responses to mild stress [136,137]. So far, animal studies are generally reassuring—Nrf2 overexpression and moderate enhancement of SOD2 or catalase typically confer protection without obvious downsides—but human data are still limited.

In light of these considerations, gene-based antioxidant therapies are best envisioned as targeted interventions for severe or refractory disease phenotypes, rather than as tools for generalized population-wide prevention in the near term [28]. They may become useful future adjuncts in cases where conventional measures and nutraceuticals are insufficient. Continued advancements in vector engineering and delivery—including improved AAV capsids and optimized delivery routes—could mitigate some safety concerns and make these therapies more feasible [28,133].

### 6.4. Synergy with Natural Antioxidants and Lifestyle Interventions

In an ideal schema, gene therapy would integrate into a layered model of prevention and treatment [28]. Lifestyle and diet establish the baseline redox resilience of an individual. Nutraceuticals and supplements can fine-tune specific pathways in those who need extra support. Then, engineered redox modulation via gene therapy addresses the most severe, tissue-specific oxidative challenges (for example, the very high oxidative stress in a failing heart or in a neurodegenerative focus) [126,138]. Rather than replacing lifestyle medicine, gene therapy would complement it by filling gaps where dietary strategies might be insufficient.

There is also potential for direct synergy between gene-based and nutritional approaches. For instance, if one uses gene therapy to upregulate Nrf2 or PGC-1α in a tissue, providing dietary polyphenols like sulforaphane or curcumin concurrently could further stabilize and activate Nrf2, enhancing transcription of antioxidant enzymes [43,121]. In this way, the combination of gene delivery and natural Nrf2 activators might yield a greater effect than either alone—a form of therapeutic synergy. Another example: gene therapy might induce increased levels of antioxidant enzyme proteins, while high-antioxidant diets supply the necessary micronutrient cofactors (e.g., selenium for glutathione peroxidase, copper/zinc for SOD1) to ensure optimal enzyme function [120].

To illustrate, consider a patient with an inherited mutation causing heart failure driven by oxidative stress (such as a cardiomyopathic form of muscular dystrophy). A future treatment might use an AAV vector to deliver SOD2 to the heart and skeletal muscle [27,126]. Concurrently, the patient would be advised to follow an antioxidant-rich diet—perhaps supplemented with specific polyphenols—to maximize endogenous antioxidant responses [121]. The gene therapy would provide a new baseline of defense, while diet and lifestyle would continue to minimize ongoing oxidative insults. This multi-layered approach could support more sustained maintenance of redox homeostasis in chronic disease states [138].

In summary, gene therapy directed toward oxidative stress is inherently complementary to lifestyle interventions. Both aim to strengthen the same endogenous systems (Nrf2 pathway, antioxidant enzymes, mitochondrial quality control). The combination promises a more robust and sustained effect: lifestyle change creates a favorable internal environment, whereas gene therapy adds an extra layer of defensive capacity. This layered strategy aligns with the broader vision presented in this review—a comprehensive redox-based framework for preventing and managing chronic diseases [28].

It should also be noted that enhancing antioxidant defenses via gene therapy is relevant not only for common diseases but also for certain rare hereditary syndromes. For example, Friedreich’s ataxia—a rare disorder caused by frataxin deficiency—features pronounced oxidative stress and reduced Nrf2/PGC-1α activity; preclinical work with AAV-based frataxin gene therapy has shown reversal of cardiomyopathy and prolonged survival in mouse models [26,126]. Similarly, some mitochondrial myopathies caused by mutations in oxidative phosphorylation genes might benefit from gene constructs encoding antioxidant proteins, helping counteract ROS-mediated muscle damage [138]. Nevertheless, the primary focus of antioxidant gene therapy development remains on prevalent chronic diseases (e.g., atherosclerosis, heart failure, neurodegeneration), where these high-tech interventions would integrate into a comprehensive strategy that also includes anti-inflammatory drugs, dietary measures, and conventional antioxidants [28,125].

At present, antioxidant-oriented gene therapy remains largely preclinical. Its inclusion in this review reflects its mechanistic continuity with redox biology rather than established clinical applicability.

## 7. The “One Ring to Rule Them All” Framework—Integrative Perspective

We propose a practical, translational “one-ring” model in which redox biology—the dynamic balance of oxidative stress, inflammation, metabolic signaling, mitochondrial quality control, and cellular senescence/SASP—serves as the shared mechanistic hub linking diverse diseases [16]. Importantly, the inclusion of rare (orphan) diseases within this framework does not imply equivalence in prevalence but rather reflects their value as high-intensity mechanistic models. In such disorders, oxidative stress and mitochondrial dysfunction are often amplified and more easily observable, thereby reinforcing the causal role of redox imbalance that may operate at lower amplitude in common chronic diseases. In this framework:

- Primary lever (population scale). Whole-food, antioxidant-rich dietary patterns and lifestyle interventions reduce chronic redox–inflammatory activation across the population [4,12]. This is the foundational ring, as lifestyle medicine is widely applicable and relatively low-cost.
- Adjunct precision tools (selected cases). Targeted nutraceuticals and supplements may refine pathway modulation in high-risk or symptomatic individuals. This layer is personalized—for example, adding omega-3 for someone with high residual inflammation, or extra coQ10 for an older patient on a statin—to address specific imbalances [33,99].
- Clinical exemplar (cardiovascular). Myocardial ischemia/reperfusion injury (as in heart attack treatment) illustrates how ROS and inflammatory cascades drive acute and chronic outcomes, and why phytochemicals and antioxidant-oriented strategies remain relevant in acute care and secondary prevention [20,21,93]. The lessons from cardioprotection studies inform us how we might tackle other acute-on-chronic oxidative injuries.
- Stress test (orphan diseases). Rare disorders reveal the extreme end of redox pathology and can generate mechanistic insights transferable to aging-related chronic disease. Orphan diseases serve as high-intensity models to validate targets (like Nrf2 or SOD) that could be harnessed in more common diseases at a lower intensity [22,105,123].
- Future layer (engineered interventions). Gene-based enhancement of endogenous defenses offers a conceptual extension for severe, tissue-specific oxidative injury. This futuristic ring would encompass gene therapies, RNA-based treatments, or gene editing techniques designed to amplify antioxidant networks in vivo [28,126].

**Figure 2:**
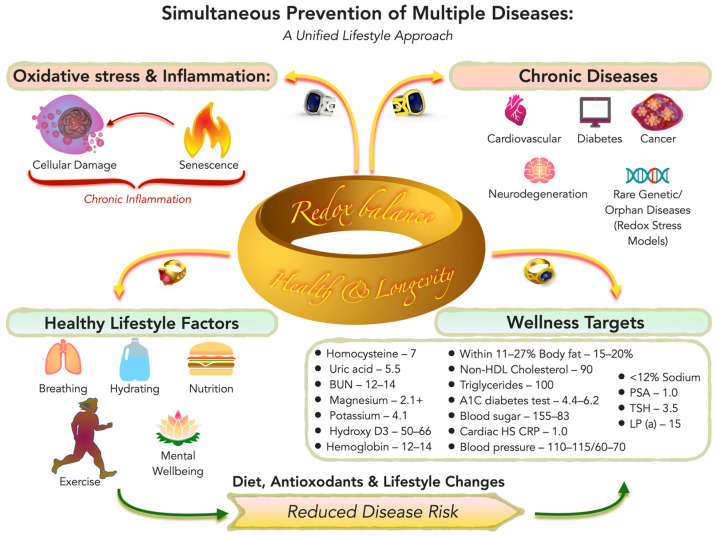
Conceptual “one-ring” framework for the simultaneous prevention of multiple chronic diseases. Central redox balance integrates oxidative stress, chronic inflammation, metabolic signaling, mitochondrial function, and cellular senescence as shared mechanistic drivers underlying cardiovascular, metabolic, malignant, neurodegenerative, and age-associated conditions. Rare (orphan) disorders characterized by extreme redox imbalance are included as high-intensity mechanistic models that reinforce the unifying role of oxidative stress. The figure represents a literature-informed conceptual synthesis rather than guideline-recommended targets.

In addition to the qualitative “one-ring” framework, Figure 2 incorporates an expert-informed, literature-based conceptual synthesis of biomarker domains that tend to cluster in individuals with the lowest observed long-term disease risk. This synthesis was supported by AI-assisted harmonization of epidemiological, interventional, and mechanistic literature to organize recurring patterns across studies. Importantly, the figure does not propose guideline-recommended targets or validated treatment thresholds. Instead, it serves as a hypothesis-generating visualization illustrating how coordinated improvements across multiple cardiometabolic and inflammatory biomarkers may reflect restoration of systemic redox balance and reduced chronic inflammation [16]. Unlike Figure 1, which presents a directional cascade, Figure 2 illustrates the integrative relationships among domains without implying equal causal weighting.

We visualize this framework as a concentric model—conceptualized as a concentric systems model, in which redox biology functions as a central integrative hub linking multiple regulatory domains. Around it are four interacting intervention layers: Lifestyle/Diet, Nutraceuticals, Cardiovascular Exemplar, and Gene-Based Modulation. Each of these points inward, influencing the central redox hub, and outward, affecting multiple disease endpoints (cardiovascular disease, diabetes, cancer risk, neurodegeneration, frailty, etc.) [4,12]. The idea is that by targeting the redox hub through various means, we can simultaneously move the needles on many diseases of aging.

A graphical abstract could depict this: a central circle labeled “Redox–Inflammation–Senescence Network” with arrows connecting out to icons of different diseases. Surrounding the center are four rings or arrows representing the layers listed above, all feeding into the center. This conveys that whether it is eating more vegetables, taking a curcumin supplement, conditioning the heart against reperfusion injury, or future gene therapy—all these actions ultimately converge on enhancing redox balance and resilience, which in turn yields multi-disease preventive effects [121].

### Quantifiable Redox-Associated Wellness Metrics

Beyond qualitative lifestyle principles, quantifiable biomarker domains may serve as operational indicators of systemic redox resilience. Clustering of favorable cardiometabolic and inflammatory markers—including low non-HDL cholesterol (e.g., <90 mg/dL), triglycerides <100 mg/dL, high-sensitivity C-reactive protein ≤1.0 mg/L, normotensive blood pressure ranges (≈110–115/60–70 mmHg), and low-normal glycemic indices—has been consistently associated with markedly reduced long-term cardiovascular and metabolic risk across epidemiological and interventional studies [9,139,140].

These values are not proposed as rigid therapeutic mandates, but rather as integrative physiologic reference zones reflecting coordinated attenuation of oxidative stress, inflammatory signaling, and metabolic overload. When multiple biomarkers simultaneously occupy low-risk ranges, they may reflect a biomarker constellation consistent with improved redox and inflammatory balance, although causality cannot be inferred from clustering alone.

## 8. Conclusions and Future Directions

The simultaneous prevention of multiple chronic diseases is a plausible and increasingly evidence-supported goal because many of these conditions share common upstream drivers—notably oxidative stress and inflammation [16]. By intervening on these shared mechanisms, we can influence a broad spectrum of disease outcomes (Table 2). The most actionable and scalable approach is lifestyle medicine, with a primary emphasis on dietary patterns rich in natural antioxidants within whole foods, alongside other key health behaviors (physical activity, not smoking, adequate sleep, etc.). These lifestyle measures establish the baseline for robust endogenous defenses and low chronic inflammation, as observed in populations largely free of Western diseases [4,12].

Targeted nutraceuticals may provide additional benefit in selected contexts as secondary levers. For example, concentrated polyphenols or omega-3 fatty acids may be used in patients with residual inflammatory risk despite a good diet, or in those with genetic susceptibilities that warrant extra intervention [33,121]. Such supplements should be used judiciously and personalized to individual needs, complementing but not substituting for the foundation of nutrition.

Our cardiovascular case study—ischemia/reperfusion injury—demonstrates the clinical importance of ROS-driven pathology and the continuing relevance of phytochemicals and antioxidant-oriented interventions even in high-tech acute care scenarios [20,141]. It reminds us that despite mixed results in some antioxidant trials, the principle of redox modulation remains vital, especially when the right therapy is applied with appropriate timing.

Orphan diseases highlight the extremes of oxidative stress and offer mechanistic lessons that likely generalize to more common age-related conditions [22,24]. The successes (and limitations) of antioxidants in these rare settings can guide how we approach complex chronic diseases: they underscore the value of combination therapy and early intervention in the disease process, and they reinforce that oxidative stress is indeed a fundamental foe across biological systems.

Finally, redox-oriented gene therapy represents a future frontier—a potent, though still experimental, layer that could complement nutrition-based strategies by directly enhancing endogenous antioxidant defenses in a tissue-specific manner for high-burden scenarios [28,126]. While not yet ready for routine clinical use, this approach illustrates a broader point: as our scientific toolkit expands, we are gaining more ways to bolster the human body’s resilience against oxidative and inflammatory damage [138].

Together, these elements support a unified redox framework for multi-disease prevention for health and longevity. By viewing diverse diseases through the common lens of redox biology and intervening accordingly—primarily with diet and lifestyle, supported by targeted nutraceuticals, and eventually advanced therapies—we can move toward a paradigm of true poly-disease prevention. In the coming years, interdisciplinary research should continue to refine this framework—for instance, by identifying optimal combinations of lifestyle and pharmacological interventions, developing biomarkers to measure an individual’s redox-inflammatory status, and conducting trials that track multiple disease endpoints rather than single outcomes. If supported by rigorous prospective validation, such approaches may contribute to meaningful reductions in chronic disease burden and improvements in population healthspan.

## Figures and Tables

**Table 1:** Summary of key sections, mechanistic focus, representative evidence, and translational implications.

Section	Core Concept	Key Mechanisms	Representative Evidence Type	Translational Implication
1. Introduction	Simultaneous prevention through lifestyle modulation	Redox–inflammation axis; gene–environment interaction	Lifestyle Heart Trial [10,12,16]; LDL meta-analyses; WHO data	Coordinated lifestyle modification reduces multiple disease risks
2. Shared Redox Mechanisms	Common upstream drivers of chronic disease	Oxidative stress; NF-κB; SASP; mTOR/AMPK imbalance; mitochondrial dysfunction; dysbiosis	Mechanistic reviews [15,16,17,18,19]	Targeting the redox network may impact multiple diseases simultaneously
3. Lifestyle & Diet	Plant-predominant dietary patterns as scalable intervention	Polyphenols; Nrf2 activation; improved NO bioavailability; microbiome modulation	Mediterranean diet data; CARDIA study; EAT-Lancet Commission [4,12]	Diet acts as a first-line, population-scale redox intervention
4. Cardiovascular Exemplar	Ischemia/reperfusion as a redox stress model	ROS burst; mitochondrial permeability transition; ferroptosis; NF-κB; Ca^2+^ overload	Experimental I/R models; resveratrol, curcumin, quercetin studies [20,21]	Demonstrates real-world relevance of redox modulation
5. Orphan Diseases	Rare diseases as high-intensity redox models	Lysosomal dysfunction; mitochondrial ROS; impaired autophagy	NPC [22], TSD [23,24], MELAS [25], Friedreich’s ataxia [26] models	Extreme models validate oxidative stress as a central driver
6. Gene-Based Modulation	Engineered reinforcement of antioxidant defenses	SOD, catalase, Nrf2, PGC-1α gene delivery	AAV-based antioxidant gene studies [27,28]	Potential future adjunct for refractory disease
7. Integrative Framework	“One-ring” redox hub model	Coordinated biomarker shifts reflect improved redox state	Conceptual synthesis + literature harmonization [4,5]	Multi-layer intervention model
8. Conclusions	Lifestyle-first, adjunctive precision tools	Redox resilience as a prevention paradigm	Epidemiology + mechanistic integration	Roadmap for multi-disease prevention

**Table 2:** Representative natural antioxidants and redox-modulating strategies: mechanisms, evidence level, and limitations.

Intervention	Primary Mechanism	Evidence Type	Representative Dose (Studied)	Typical Duration	Limitations
Resveratrol	Endothelial nitric oxide synthase (eNOS) activation; Nrf2 induction; mitochondrial protection	Animal I/R models; mechanistic studies	10–50 mg/kg (rodent models); human data variable [21,91]	1–4 weeks (preclinical)	Low oral bioavailability; inconsistent clinical efficacy
Curcumin	NF-κB inhibition; Nrf2 activation; anti-inflammatory remodeling	Animal MI models; limited human data	100–200 mg/kg (animal); 500–2000 mg/day (human studies) [92,93]	4–8 weeks (typical trial range)	Poor absorption; formulation-dependent effects
Quercetin	Lipid peroxidation inhibition; mitochondrial stabilization	Rodent I/R models	10–50 mg/kg (animal models) [94,95]	1–4 weeks	Limited robust human outcome trials
Green tea catechins	Phase 2 enzyme induction; antioxidant enzyme upregulation	Nutritional intervention studies; mechanistic models	400–800 mg epigallocatechin gallate (EGCG)/day (clinical range) [96]	2–12 weeks	Heterogeneity in extracts; dose variability
Coenzyme Q10 (coQ10)	Mitochondrial electron transport support; lipid-phase antioxidant	Q-SYMBIO randomized controlled trial (RCT)	100 mg three times a day [99]	2 years	Modest effect size; patient selection is important
Alpha-lipoic acid	Glutathione regeneration; phosphatidylinositol 3-kinase (PI3K)/protein kinase B (Akt)/Nrf2 pathway	Rodent I/R studies; small human trials	50–100 mg/kg (animal); 300–600 mg/day (human) [100,101]	Variable (weeks–months)	Limited large-scale cardiovascular RCTs
Anthocyanin-rich berries	Endothelial protection; Nrf2 signaling; vascular antioxidant effects	Human and preclinical vascular studies	160–500 mg anthocyanins/day (typical supplementation studies) [97]	4–12 weeks	Limited long-term outcome data
Omega-3 fatty acids	Anti-inflammatory lipid mediators	Large RCTs; meta-analyses	1–4 g/day eicosapentaenoic acid (EPA)/docosahexaenoic acid (DHA) [102]	1–5 years (RCTs)	Mixed outcomes across populations
Selenium (deficiency correction)	Glutathione peroxidase cofactor; redox enzyme support	Deficiency correction studiess; mechanistic models	Dose individualized to plasma selenium status [103,104]	Variable	Narrow therapeutic window; benefit mainly in deficiency
N-acetylcysteine (NAC)	Glutathione precursor; ROS buffering	Mitochondrial disease models; limited human data	600–1800 mg/day (clinical contexts) [105]	Variable	Limited chronic disease RCT data
Antioxidant gene therapy (SOD, catalase, Nrf2)	Direct ROS neutralization via transgene expression	Preclinical models	Vector-dependent (AAV delivery) [27,106]	Single or limited dosing	Delivery, safety, and durability challenges

## Data Availability

The original contributions presented in this study are included in the article. Further inquiries can be directed to the corresponding author.

## Abbreviations

The following abbreviations are used in this manuscript:

AAV	Adeno-associated virus
Akt	Protein kinase B
AMPK	AMP-activated protein kinase
CNS	Central nervous system
coQ10	Coenzyme Q10
DHA	Docosahexaenoic acid
EGCG	Epigallocatechin gallate
eNOS	Endothelial nitric oxide synthase
EPA	Eicosapentaenoic acid
GPX	Glutathione peroxidases
HO	Heme oxygenase
I/R	Ischemia/reperfusion
LDL	Low-density lipoprotein
LSD	Lysosomal storage disease
MI	Myocardial infarction
mTOR	Mechanistic target of rapamycin
NAC	N-acetylcysteine
NF-κB	Nuclear factor kappa-light-chain-enhancer of activated B cells
NPC	Niemann–Pick disease type C
NPD	Niemann–Pick disease
Nrf2	Nuclear factor erythroid 2-related factor 2
PGC-1α	Peroxisome proliferator-activated receptor gamma coactivator 1-alpha
PI3K	Phosphatidylinositol 3-kinase
RII	Reperfusion-induced injury
ROS	Reactive oxygen species
SASP	Senescence-associated secretory phenotype
SCFA	Short-chain fatty acid
SOD	Superoxide dismutase
TMAO	Trimethylamine N-oxide
TSD	Tay–Sachs disease

## References

[1] Magnussen C., Alegre-Diaz J., Al-Nasser L.A., Amouyel P., Aviles-Santa L., Bakker S.J.L., Ballantyne C.M., Bernabe-Ortiz A., Bobak M., The Global Cardiovascular Risk Consortium (2025). Global Effect of Cardiovascular Risk Factors on Lifetime Estimates. N. Engl. J. Med..

[2] Virani S.S., Alonso A., Aparicio H.J., Benjamin E.J., Bittencourt M.S., Callaway C.W., Carson A.P., Chamberlain A.M., Cheng S., Delling F.N. (2021). Heart Disease and Stroke Statistics-2021 Update: A Report From the American Heart Association. Circulation.

[3] Giri J., Olin J.W. (2019). The CREST-2 Registry: First Glimpse at a Carotid Stenting Comeback?. J. Am. Coll. Cardiol..

[4] Willett W., Rockstrom J., Loken B., Springmann M., Lang T., Vermeulen S., Garnett T., Tilman D., DeClerck F., Wood A. (2019). Food in the Anthropocene: The EAT-Lancet Commission on healthy diets from sustainable food systems. Lancet.

[5] Hull S.C., Mszar R., Ostfeld R.J., Ferrucci L.M., Mucci L.A., Giovannucci E., Loeb S. (2025). Diet and Prevention of Cardiovascular Disease and Cancer: JACC: CardioOncology State-of-the-Art Review. JACC CardioOnco..

[6] Robert Silverstein H. (2003). National Cholesterol Education Program Adult Treatment Panel-III guidelines and the abolition of symptomatic coronary artery disease. Am. J. Cardiol..

[7] Robert Silverstein H. (2008). Re: “Call to action on use and reimbursement for home blood pressure monitoring: Executive summary”. J. Clin. Hypertens..

[8] Cholesterol Treatment Trialists C., Baigent C., Blackwell L., Emberson J., Holland L.E., Reith C., Bhala N., Peto R., Barnes E.H., Keech A. (2010). Efficacy and safety of more intensive lowering of LDL cholesterol: A meta-analysis of data from 170,000 participants in 26 randomised trials. Lancet.

[9] Ference B.A., Ginsberg H.N., Graham I., Ray K.K., Packard C.J., Bruckert E., Hegele R.A., Krauss R.M., Raal F.J., Schunkert H. (2017). Low-density lipoproteins cause atherosclerotic cardiovascular disease. 1. Evidence from genetic, epidemiologic, and clinical studies. A consensus statement from the European Atherosclerosis Society Consensus Panel. Eur. Heart J..

[10] Ornish D., Brown S.E., Scherwitz L.W., Billings J.H., Armstrong W.T., Ports T.A., McLanahan S.M., Kirkeeide R.L., Brand R.J., Gould K.L. (1990). Can lifestyle changes reverse coronary heart disease? The Lifestyle Heart Trial. Lancet.

[11] Pickering T.G., Miller N.H., Ogedegbe G., Krakoff L.R., Artinian N.T., Goff D., American Heart Association, American Society of Hypertension, Preventive Cardiovascular Nurses Association (2008). Call to action on use and reimbursement for home blood pressure monitoring: Executive summary: A joint scientific statement from the American Heart Association, American Society Of Hypertension, and Preventive Cardiovascular Nurses Association. Hypertension.

[12] Ornish D., Scherwitz L.W., Billings J.H., Brown S.E., Gould K.L., Merritt T.A., Sparler S., Armstrong W.T., Ports T.A., Kirkeeide R.L. (1998). Intensive lifestyle changes for reversal of coronary heart disease. JAMA.

[13] Temba G.S., Pecht T., Kullaya V.I., Vadaq N., Mosha M.V., Ulas T., Kanungo S., van Emst L., Bonaguro L., Schulte-Schrepping J. (2025). Immune and metabolic effects of African heritage diets versus Western diets in men: A randomized controlled trial. Nat. Med..

[14] Chronic Disease Fast Facts: Health and Economic Costs of Chronic Conditions. https://www.cdc.gov/chronic-disease/data-research/facts-stats/index.html.

[15] Haines D.D., Juhasz B., Tosaki A. (2013). Management of multicellular senescence and oxidative stress. J. Cell Mol. Med..

[16] Franceschi C., Garagnani P., Vitale G., Capri M., Salvioli S. (2017). Inflammaging and ‘Garb-aging’. Trends Endocrinol. Metab..

[17] Saxton R.A., Sabatini D.M. (2017). mTOR Signaling in Growth, Metabolism, and Disease. Cell.

[18] Hardie D.G., Ross F.A., Hawley S.A. (2012). AMPK: A nutrient and energy sensor that maintains energy homeostasis. Nat. Rev. Mol. Cell Biol..

[19] Tang W.H.W., Li D.Y., Hazen S.L. (2019). Dietary metabolism, the gut microbiome, and heart failure. Nat. Rev. Cardiol..

[20] Connelly C.M., Vogel W.M., Wiegner A.W., Osmers E.L., Bing O.H., Kloner R.A., Dunn-Lanchantin D.M., Franzblau C., Apstein C.S. (1985). Effects of reperfusion after coronary artery occlusion on post-infarction scar tissue. Circ. Res..

[21] Hung L.M., Su M.J., Chen J.K. (2004). Resveratrol protects myocardial ischemia-reperfusion injury through both NO-dependent and NO-independent mechanisms. Free Radic. Biol. Med..

[22] Lee D., Hong J.H. (2023). Niemann-Pick Disease Type C (NPDC) by Mutation of NPC1 and NPC2: Aberrant Lysosomal Cholesterol Trafficking and Oxidative Stress. Antioxidants.

[23] Shaimardanova A.A., Chulpanova D.S., Solovyeva V.V., Garanina E.E., Salafutdinov I.I., Laikov A.V., Kursenko V.V., Chakrabarti L., Zakharova E.Y., Bukina T.M. (2021). Serum Cytokine Profile, Beta-Hexosaminidase A Enzymatic Activity and GM_2_ Ganglioside Levels in the Plasma of a Tay-Sachs Disease Patient after Cord Blood Cell Transplantation and Curcumin Administration: A Case Report. Life.

[24] Solovyeva V.V., Shaimardanova A.A., Chulpanova D.S., Kitaeva K.V., Chakrabarti L., Rizvanov A.A. (2018). New Approaches to Tay-Sachs Disease Therapy. Front. Physiol..

[25] Garrido-Maraver J., Cordero M.D., Monino I.D., Pereira-Arenas S., Lechuga-Vieco A.V., Cotan D., De la Mata M., Oropesa-Avila M., De Miguel M., Bautista Lorite J. (2012). Screening of effective pharmacological treatments for MELAS syndrome using yeasts, fibroblasts and cybrid models of the disease. Br. J. Pharmacol..

[26] Sivakumar A., Cherqui S. (2022). Advantages and Limitations of Gene Therapy and Gene Editing for Friedreich’s Ataxia. Front. Genome Ed..

[27] Li Q., Bolli R., Qiu Y., Tang X.L., Guo Y., French B.A. (2001). Gene therapy with extracellular superoxide dismutase protects conscious rabbits against myocardial infarction. Circulation.

[28] Levonen A.L., Vahakangas E., Koponen J.K., Yla-Herttuala S. (2008). Antioxidant gene therapy for cardiovascular disease: Current status and future perspectives. Circulation.

[29] Sies H. (2015). Oxidative stress: A concept in redox biology and medicine. Redox Biol..

[30] Sen C.K., Packer L. (1996). Antioxidant and redox regulation of gene transcription. FASEB J..

[31] Forman H.J., Zhang H. (2021). Targeting oxidative stress in disease: Promise and limitations of antioxidant therapy. Nat. Rev. Drug Discov..

[32] Tosti V., Bertozzi B., Fontana L. (2018). Health Benefits of the Mediterranean Diet: Metabolic and Molecular Mechanisms. J. Gerontol. A Biol. Sci. Med. Sci..

[33] Joshi S., McMacken M., Kalantar-Zadeh K. (2021). Plant-Based Diets for Kidney Disease: A Guide for Clinicians. Am. J. Kidney Dis..

[34] Packer M. (2025). The Adipokine Hypothesis of Heart Failure with a Preserved Ejection Fraction: A Novel Framework to Explain Pathogenesis and Guide Treatment. J. Am. Coll. Cardiol..

[35] Coppe J.P., Desprez P.Y., Krtolica A., Campisi J. (2010). The senescence-associated secretory phenotype: The dark side of tumor suppression. Annu. Rev. Pathol..

[36] Gorodilova A.V., Kharisova C.B., Osinnikova M.N., Kitaeva K.V., Filin I.Y., Mayasin Y.P., Solovyeva V.V., Rizvanov A.A. (2024). The Well-Forgotten Old: Platelet-Rich Plasma in Modern Anti-Aging Therapy. Cells.

[37] Fernandez E., Wargo J.A., Helmink B.A. (2025). The Microbiome and Cancer: A Translational Science Review. JAMA.

[38] Gibson G.R., Hutkins R., Sanders M.E., Prescott S.L., Reimer R.A., Salminen S.J., Scott K., Stanton C., Swanson K.S., Cani P.D. (2017). Expert consensus document: The International Scientific Association for Probiotics and Prebiotics (ISAPP) consensus statement on the definition and scope of prebiotics. Nat. Rev. Gastroenterol. Hepatol..

[39] Wolfe B.E., Button J.E., Santarelli M., Dutton R.J. (2014). Cheese rind communities provide tractable systems for in situ and in vitro studies of microbial diversity. Cell.

[40] Ryu D., Mouchiroud L., Andreux P.A., Katsyuba E., Moullan N., Nicolet-Dit-Felix A.A., Williams E.G., Jha P., Lo Sasso G., Huzard D. (2016). Urolithin A induces mitophagy and prolongs lifespan in C. elegans and increases muscle function in rodents. Nat. Med..

[41] Yi S.Y., Steffen L.M., Guan W., Duprez D., Lakshminarayan K., Jacobs D.R. (2025). Dietary carbohydrate quality, fibre-rich food intake, and left ventricular structure and function: The CARDIA study. Eur. Heart J..

[42] O’Keefe S.J., Li J.V., Lahti L., Ou J., Carbonero F., Mohammed K., Posma J.M., Kinross J., Wahl E., Ruder E. (2015). Fat, fibre and cancer risk in African Americans and rural Africans. Nat. Commun..

[43] Obeme-Nmom J.I., Abioye R.O., Reyes Flores S.S., Udenigwe C.C. (2024). Regulation of redox enzymes by nutraceuticals: A review of the roles of antioxidant polyphenols and peptides. Food Funct..

[44] Heiss C., Schroeter H., Balzer J., Kleinbongard P., Matern S., Sies H., Kelm M. (2006). Endothelial function, nitric oxide, and cocoa flavanols. J. Cardiovasc. Pharmacol..

[45] Jurja S., Negreanu-Pirjol T., Mehedinti M.C., Hincu M.A., Negreanu-Pirjol B.S., Roncea F.N., Laurentiu Tatu A. (2025). Blueberries and Honeysuckle Berries: Anthocyanin-Rich Polyphenols for Vascular Endothelial Health and Cardiovascular Disease Prevention. Nutrients.

[46] Jones T., Dunn E.L., Macdonald J.H., Kubis H.P., McMahon N., Sandoo A. (2019). The Effects of Beetroot Juice on Blood Pressure, Microvascular Function and Large-Vessel Endothelial Function: A Randomized, Double-Blind, Placebo-Controlled Pilot Study in Healthy Older Adults. Nutrients.

[47] Darvish S., Ludwig K., Ikoba A., Berryman-Maciel M., Coppock M., Murray K., Chonchol M., Seals D., Rossman M. (2024). Nitrate-rich beetroot juice supplementation in midlife and older adults with renal dysfunction increases vascular endothelial function and changes the circulating milieu to improve endothelial cell nitric oxide production and oxidative stress. Physiology.

[48] Khera A.V., Emdin C.A., Drake I., Natarajan P., Bick A.G., Cook N.R., Chasman D.I., Baber U., Mehran R., Rader D.J. (2016). Genetic Risk, Adherence to a Healthy Lifestyle, and Coronary Disease. N. Engl. J. Med..

[49] Rozich E., Ozkurede U., Pakkiriswami S., Gemilere R., Azarin S.M., Liu J.C. (2025). Mitochondrial oxidative stress, calcium and dynamics in cardiac ischaemia-reperfusion injury. J. Physiol..

[50] Oerlemans M.I., Koudstaal S., Chamuleau S.A., de Kleijn D.P., Doevendans P.A., Sluijter J.P. (2013). Targeting cell death in the reperfused heart: Pharmacological approaches for cardioprotection. Int. J. Cardiol..

[51] Zweier J.L., Flaherty J.T., Weisfeldt M.L. (1987). Direct measurement of free radical generation following reperfusion of ischemic myocardium. Proc. Natl. Acad. Sci. USA.

[52] Walker M.J., Curtis M.J., Hearse D.J., Campbell R.W., Janse M.J., Yellon D.M., Cobbe S.M., Coker S.J., Harness J.B., Harron D.W. (1988). The Lambeth Conventions: Guidelines for the study of arrhythmias in ischaemia infarction, and reperfusion. Cardiovasc. Res..

[53] Hearse D.J., Tosaki A. (1988). Free radicals and calcium: Simultaneous interacting triggers as determinants of vulnerability to reperfusion-induced arrhythmias in the rat heart. J. Mol. Cell Cardiol..

[54] Li G., Chen Y., Saari J.T., Kang Y.J. (1997). Catalase-overexpressing transgenic mouse heart is resistant to ischemia-reperfusion injury. Am. J. Physiol..

[55] Shen S., He F., Cheng C., Xu B., Sheng J. (2021). Uric acid aggravates myocardial ischemia-reperfusion injury via ROS/NLRP3 pyroptosis pathway. Biomed. Pharmacother..

[56] Firoozabadi M.D., Nooralishahi B., Rezaei-Tazangi F. (2026). Oxycodone: A Pain-Relieving Agent with Cardioprotective Properties Against Myocardial Ischemia-Reperfusion Injury. Cardiovasc. Ther..

[57] Schettini A., Lippman R.H., Walsh E.K. (1989). Attenuation of decompressive hypoperfusion and cerebral edema by superoxide dismutase. J. Neurosurg..

[58] Knuckey N.W., Palm D., Primiano M., Epstein M.H., Johanson C.E. (1995). N-acetylcysteine enhances hippocampal neuronal survival after transient forebrain ischemia in rats. Stroke.

[59] Aoki T., Sumii T., Mori T., Wang X., Lo E.H. (2002). Blood-brain barrier disruption and matrix metalloproteinase-9 expression during reperfusion injury: Mechanical versus embolic focal ischemia in spontaneously hypertensive rats. Stroke.

[60] Yang J., Li L., Chen X., Cai P., Chen M., Cheng J., Ma L., Zhao X., Yang P. (2026). Research progress of isoflurane in alleviating cerebral ischemia/reperfusion injury. Eur. J. Pharmacol..

[61] Hoshino T., Maley W.R., Bulkley G.B., Williams G.M. (1988). Ablation of free radical-mediated reperfusion injury for the salvage of kidneys taken from non-heartbeating donors. A quantitative evaluation of the proportion of injury caused by reperfusion following periods of warm, cold, and combined warm and cold ischemia. Transplantation.

[62] Zhou W., Farrar C.A., Abe K., Pratt J.R., Marsh J.E., Wang Y., Stahl G.L., Sacks S.H. (2000). Predominant role for C5b-9 in renal ischemia/reperfusion injury. J. Clin. Investig..

[63] Monir N., Saber M.M., Awad A.S., Elsherbiny M.E., Zaki H.F. (2022). Repression of inflammatory pathways with Boswellia for alleviation of liver injury after renal ischemia reperfusion. Life Sci..

[64] Wang K., Wang H., Zhang Y., Zhang Z., Wang L., Yang J., Man J., Yang L. (2026). Reprogramming mitochondrial homeostasis in renal ischemia-reperfusion injury. Cell Signal.

[65] Kuratani T., Matsuda H., Sawa Y., Kaneko M., Nakano S., Kawashima Y. (1992). Experimental study in a rabbit model of ischemia-reperfusion lung injury during cardiopulmonary bypass. J. Thorac. Cardiovasc. Surg..

[66] Sommer S.P., Sommer S., Sinha B., Wiedemann J., Otto C., Aleksic I., Schimmer C., Leyh R.G. (2011). Ischemia-reperfusion injury-induced pulmonary mitochondrial damage. J. Heart Lung Transplant..

[67] Dai S., Wan X., Xia L., Xu L., Xie C., Wang G., Tang J. (2026). HIF1alpha Attenuated the Lung Ischemia-Reperfusion Injury by Activating the miR-485/Notch1 Signalling. J. Cell Mol. Med..

[68] Jaeschke H., Farhood A., Smith C.W. (1990). Neutrophils contribute to ischemia/reperfusion injury in rat liver in vivo. FASEB J..

[69] Hsu C.M., Wang J.S., Liu C.H., Chen L.W. (2002). Kupffer cells protect liver from ischemia-reperfusion injury by an inducible nitric oxide synthase-dependent mechanism. Shock.

[70] Zhang X.J., Cheng X., Yan Z.Z., Fang J., Wang X., Wang W., Liu Z.Y., Shen L.J., Zhang P., Wang P.X. (2018). An ALOX12-12-HETE-GPR31 signaling axis is a key mediator of hepatic ischemia-reperfusion injury. Nat. Med..

[71] Yang Z., Hu Y., Yang C., Li Y., Zhong X., Wu Z., Zuo Y., Gan S., Chen L., Zeng Z. (2026). SMYD2-mediated methylation of STAT1 protects against hepatic ischaemia/reperfusion injury by blocking JAK-STAT1 signalling pathways. Gut.

[72] Horton J.W., White D.J. (1991). Cardiac contractile injury after intestinal ischemia-reperfusion. Am. J. Physiol..

[73] Hoehn R.S., Seitz A.P., Jernigan P.L., Gulbins E., Edwards M.J. (2016). Ischemia/Reperfusion Injury Alters Sphingolipid Metabolism in the Gut. Cell Physiol. Biochem..

[74] Pi Y., Wang Y., Guo Q., Zheng W., Zhou H., Deng L., Xu N., Song H. (2026). Oleanolic acid alleviates intestinal injury after hepatic ischemia-reperfusion under steatosis via PPARG-dependent M2 macrophage polarization. Int. Immunopharmacol..

[75] Kopacz M., Karwatowska-Prokopczuk E., Beresewicz A. (1993). Reperfusion arrhythmias and purine wash-out in isolated rat and rabbit heart. Effect of allopurinol, dimethylthiourea and calcium reduction. J. Mol. Cell Cardiol..

[76] Mendoza A., Patel P., Robichaux D., Ramirez D., Karch J. (2024). Inhibition of the mPTP and Lipid Peroxidation Is Additively Protective Against I/R Injury. Circ. Res..

[77] Jennings R.B., Reimer K.A., Steenbergen C. (1986). Myocardial ischemia revisited. The osmolar load, membrane damage, and reperfusion. J. Mol. Cell Cardiol..

[78] Curtis M.J., Hearse D.J. (1989). Reperfusion-induced arrhythmias are critically dependent upon occluded zone size: Relevance to the mechanism of arrhythmogenesis. J. Mol. Cell Cardiol..

[79] Yamada M., Hearse D.J., Curtis M.J. (1990). Reperfusion and readmission of oxygen. Pathophysiological relevance of oxygen-derived free radicals to arrhythmogenesis. Circ. Res..

[80] Dong Q., Zhu Y., Zhang X., Li L., Yang Y., Liu C., Wen J. (2025). Phytochemicals Targeting Mitophagy to Treat Heart Diseases: Retrospective Insights and Prospective Directions. Phytother. Res..

[81] Wang X., Chen T., Chen S., Zhang J., Cai L., Liu C., Zhang Y., Wu X., Li N., Ma Z. (2025). STING aggravates ferroptosis-dependent myocardial ischemia-reperfusion injury by targeting GPX4 for autophagic degradation. Signal Transduct. Target. Ther..

[82] Gong Y., Yang H., Chen T., Zhang J., Kong B., Shuai W., Huang H. (2025). USP38 exacerbates myocardial injury and malignant ventricular arrhythmias after ischemia/reperfusion by promoting ferroptosis through the P53/SLC7A11 pathway. Int. Immunopharmacol..

[83] Wen J., Li L., Yang Y., Ou D., Yang J., Xie J., Du W., Tong Y. (2024). Phytochemicals targeting ferroptosis in cardiovascular diseases: Recent advances and therapeutic perspectives. Phytother. Res..

[84] Huang F., Yang R., Xiao Z., Xie Y., Lin X., Zhu P., Zhou P., Lu J., Zheng S. (2021). Targeting Ferroptosis to Treat Cardiovascular Diseases: A New Continent to Be Explored. Front. Cell Dev. Biol..

[85] Chen Y.F., Chen W.Y., Chung C.H., Kuo C.L., Lee A.S. (2020). Cardiac protection of Bauhinia championii against reperfusion injury. Environ. Toxicol..

[86] Szobi A., Farkasova-Ledvenyiova V., Lichy M., Murarikova M., Carnicka S., Ravingerova T., Adameova A. (2018). Cardioprotection of ischaemic preconditioning is associated with inhibition of translocation of MLKL within the plasma membrane. J. Cell Mol. Med..

[87] Li T., Wang N., Yi D., Xiao Y., Li X., Shao B., Wu Z., Bai J., Shi X., Wu C. (2025). ROS-mediated ferroptosis and pyroptosis in cardiomyocytes: An update. Life Sci..

[88] Maslov L.N., Popov S.V., Naryzhnaya N.V., Mukhomedzyanov A.V., Kurbatov B.K., Derkachev I.A., Boshchenko A.A., Prasad N.R., Ma H., Zhang Y. (2023). K(ATP) channels are regulators of programmed cell death and targets for the creation of novel drugs against ischemia/reperfusion cardiac injury. Fundam. Clin. Pharmacol..

[89] Ji N., Qi Z., Wang Y., Yang X., Yan Z., Li M., Ge Q., Zhang J. (2021). Pyroptosis: A New Regulating Mechanism in Cardiovascular Disease. J. Inflamm. Res..

[90] Koshinuma S., Miyamae M., Kaneda K., Kotani J., Figueredo V.M. (2014). Combination of necroptosis and apoptosis inhibition enhances cardioprotection against myocardial ischemia-reperfusion injury. J. Anesth..

[91] Xia N., Forstermann U., Li H. (2014). Resveratrol and endothelial nitric oxide. Molecules.

[92] Wang N.P., Wang Z.F., Tootle S., Philip T., Zhao Z.Q. (2012). Curcumin promotes cardiac repair and ameliorates cardiac dysfunction following myocardial infarction. Br. J. Pharmacol..

[93] Zeng C., Zhong P., Zhao Y., Kanchana K., Zhang Y., Khan Z.A., Chakrabarti S., Wu L., Wang J., Liang G. (2015). Curcumin protects hearts from FFA-induced injury by activating Nrf2 and inactivating NF-kappaB both in vitro and in vivo. J. Mol. Cell Cardiol..

[94] Liu H., Zhang L., Lu S. (2012). Evaluation of antioxidant and immunity activities of quercetin in isoproterenol-treated rats. Molecules.

[95] Brookes P.S., Digerness S.B., Parks D.A., Darley-Usmar V. (2002). Mitochondrial function in response to cardiac ischemia-reperfusion after oral treatment with quercetin. Free Radic. Biol. Med..

[96] Akhlaghi M., Bandy B. (2010). Dietary green tea extract increases phase 2 enzyme activities in protecting against myocardial ischemia-reperfusion. Nutr. Res..

[97] Najjar R.S., Schwartz A.M., Wong B.J., Mehta P.K., Feresin R.G. (2021). Berries and Their Polyphenols as a Potential Therapy for Coronary Microvascular Dysfunction: A Mini-Review. Int. J. Mol. Sci..

[98] Ablon G. (2021). Nutraceuticals. Dermatol. Clin..

[99] Mortensen S.A., Rosenfeldt F., Kumar A., Dolliner P., Filipiak K.J., Pella D., Alehagen U., Steurer G., Littarru G.P., Investigators Q.S.S. (2014). The effect of coenzyme Q10 on morbidity and mortality in chronic heart failure: Results from Q-SYMBIO: A randomized double-blind trial. JACC Heart Fail..

[100] Qi B., Zheng Y., Gao W., Qi Z., Gong Y., Liu Y., Wang Y., Cheng X., Ning M., Lang Y. (2022). Alpha-lipoic acid impedes myocardial ischemia-reperfusion injury, myocardial apoptosis, and oxidative stress by regulating HMGB1 expression. Eur. J. Pharmacol..

[101] Deng C., Sun Z., Tong G., Yi W., Ma L., Zhao B., Cheng L., Zhang J., Cao F., Yi D. (2013). α-Lipoic acid reduces infarct size and preserves cardiac function in rat myocardial ischemia/reperfusion injury through activation of PI3K/Akt/Nrf2 pathway. PLoS ONE.

[102] Bhatt D.L., Steg P.G., Miller M., Brinton E.A., Jacobson T.A., Ketchum S.B., Doyle R.T., Juliano R.A., Jiao L., Granowitz C. (2019). Cardiovascular Risk Reduction with Icosapent Ethyl for Hypertriglyceridemia. N. Engl. J. Med..

[103] Rayman M.P. (2012). Selenium and human health. Lancet.

[104] Mendelev N., Mehta S.L., Idris H., Kumari S., Li P.A. (2012). Selenite stimulates mitochondrial biogenesis signaling and enhances mitochondrial functional performance in murine hippocampal neuronal cells. PLoS ONE.

[105] Polyak E., Ostrovsky J., Peng M., Dingley S.D., Tsukikawa M., Kwon Y.J., McCormack S.E., Bennett M., Xiao R., Seiler C. (2018). N-acetylcysteine and vitamin E rescue animal longevity and cellular oxidative stress in pre-clinical models of mitochondrial complex I disease. Mol. Genet. Metab..

[106] Zhu H.L., Stewart A.S., Taylor M.D., Vijayasarathy C., Gardner T.J., Sweeney H.L. (2000). Blocking free radical production via adenoviral gene transfer decreases cardiac ischemia-reperfusion injury. Mol. Ther..

[107] Tan H.H., Liang Y.C., Shao Y.C., Chen C.M., Chou W. (2025). Impact of selenium status and supplementation on outcomes in critically ill patients. Sci. Rep..

[108] Bano I., Hassan M.F., Kieliszek M. (2025). A Comprehensive Review of Selenium as a Key Regulator in Thyroid Health. Biol. Trace Elem. Res..

[109] Basirli H., Ates N., Seyrantepe V. (2025). Imbalance in redox homeostasis is associated with neurodegeneration in the murine model of Tay-Sachs disease. Mol. Biol. Rep..

[110] Bar S., Prasad M., Datta R. (2018). Neuromuscular degeneration and locomotor deficit in a Drosophila model of mucopolysaccharidosis VII is attenuated by treatment with resveratrol. Dis. Model. Mech..

[111] Lee Y.J., Kim S.J., Heo T.H. (2011). Protective effect of catechin in type I Gaucher disease cells by reducing endoplasmic reticulum stress. Biochem. Biophys. Res. Commun..

[112] Williams I.M., Wallom K.L., Smith D.A., Al Eisa N., Smith C., Platt F.M. (2014). Improved neuroprotection using miglustat, curcumin and ibuprofen as a triple combination therapy in Niemann-Pick disease type C1 mice. Neurobiol. Dis..

[113] Klemmensen M.M., Borrowman S.H., Pearce C., Pyles B., Chandra B. (2024). Mitochondrial dysfunction in neurodegenerative disorders. Neurotherapeutics.

[114] Souza D.S., Barreto T.O., Menezes-Filho J.E.R., Heimfarth L., Rhana P., Rabelo T.K., Santana M.N.S., Durco A.O., Conceicao M.R.L., Quintans-Junior L.J. (2020). Myocardial hypertrophy is prevented by farnesol through oxidative stress and ERK1/2 signaling pathways. Eur. J. Pharmacol..

[115] Sitarska D., Tylki-Szymanska A., Lugowska A. (2021). Treatment trials in Niemann-Pick type C disease. Metab. Brain Dis..

[116] Guy J., Qi X., Wang H., Hauswirth W.W. (1999). Adenoviral gene therapy with catalase suppresses experimental optic neuritis. Arch. Ophthalmol..

[117] Parkinson M.H., Schulz J.B., Giunti P. (2013). Co-enzyme Q10 and idebenone use in Friedreich’s ataxia. J. Neurochem..

[118] Kearney M., Orrell R.W., Fahey M., Brassington R., Pandolfo M. (2016). Pharmacological treatments for Friedreich ataxia. Cochrane Database Syst. Rev..

[119] Mehta S.L., Kumari S., Mendelev N., Li P.A. (2012). Selenium preserves mitochondrial function, stimulates mitochondrial biogenesis, and reduces infarct volume after focal cerebral ischemia. BMC Neurosci..

[120] Jomova K., Alomar S.Y., Alwasel S.H., Nepovimova E., Kuca K., Valko M. (2024). Several lines of antioxidant defense against oxidative stress: Antioxidant enzymes, nanomaterials with multiple enzyme-mimicking activities, and low-molecular-weight antioxidants. Arch. Toxicol..

[121] Ramli N.Z., Yahaya M.F., Tooyama I., Damanhuri H.A. (2020). A Mechanistic Evaluation of Antioxidant Nutraceuticals on Their Potential against Age-Associated Neurodegenerative Diseases. Antioxidants.

[122] Liguori I., Russo G., Curcio F., Bulli G., Aran L., Della-Morte D., Gargiulo G., Testa G., Cacciatore F., Bonaduce D. (2018). Oxidative stress, aging, and diseases. Clin. Interv. Aging.

[123] Jimenez-Jimenez F.J., Alonso-Navarro H., Garcia-Martin E., Carcamo-Fonfria A., Martin-Gomez M.A., Agundez J.A.G. (2025). Oxidative Stress and Antioxidant Therapies in Friedreich’s Ataxia. Cells.

[124] Fede M.S., Daziani G., Tavoletta F., Montana A., Compagnucci P., Goteri G., Neri M., Busardo F.P. (2025). Myocardial Ischemia/Reperfusion Injury: Molecular Insights, Forensic Perspectives, and Therapeutic Horizons. Cells.

[125] Xiong Z., Liao Y., Zhang Z., Wan Z., Liang S., Guo J. (2025). Molecular Insights into Oxidative-Stress-Mediated Cardiomyopathy and Potential Therapeutic Strategies. Biomolecules.

[126] Chang J.C., Ryan M.R., Stark M.C., Liu S., Purushothaman P., Bolan F., Johnson C.A., Champe M., Meng H., Lawlor M.W. (2024). AAV8 gene therapy reverses cardiac pathology and prevents early mortality in a mouse model of Friedreich’s ataxia. Mol. Ther. Methods Clin. Dev..

[127] Kang D.H., Kang S.W. (2013). Targeting cellular antioxidant enzymes for treating atherosclerotic vascular disease. Biomol. Ther..

[128] McCord J.M. (2004). Therapeutic control of free radicals. Drug Discov. Today.

[129] Van-Assche T., Huygelen V., Crabtree M.J., Antoniades C. (2011). Gene therapy targeting inflammation in atherosclerosis. Curr. Pharm. Des..

[130] Yang Y., Zou H. (2025). Research progress on Nrf2 intervention in the treatment of diabetic retinopathy. Front. Endocrinol..

[131] Lee Y.J., Kwon S.B., An J.M., Kim C.H., Lee S.H., Choi C.Y., Nam D.H., Park J.W., Nam H.S., Lee S.H. (2015). Increased protein oxidation and decreased expression of nuclear factor E2-related factor 2 protein in skin tissue of patients with diabetes. Clin. Exp. Dermatol..

[132] Barakat M., Han C., Chen L., David B.P., Shi J., Xu A., Skowron K.J., Johnson T., Woods R.A., Ankireddy A. (2024). Non-electrophilic NRF2 activators promote wound healing in human keratinocytes and diabetic mice and demonstrate selective downstream gene targeting. Sci. Rep..

[133] Svensson E.C., Marshall D.J., Woodard K., Lin H., Jiang F., Chu L., Leiden J.M. (1999). Efficient and stable transduction of cardiomyocytes after intramyocardial injection or intracoronary perfusion with recombinant adeno-associated virus vectors. Circulation.

[134] Li Q., Bolli R., Qiu Y., Tang X.L., Murphree S.S., French B.A. (1998). Gene therapy with extracellular superoxide dismutase attenuates myocardial stunning in conscious rabbits. Circulation.

[135] Li J., Hu S.J., Sun J., Zhu Z.H., Zheng X., Wang G.Z., Yao Y.M., Chen N.Y., Zhao X.Y. (2005). Construction of phospholamban antisense RNA recombinant adeno-associated virus vector and its effects in rat cardiomyocytes. Acta Pharmacol. Sin..

[136] Zhou S., Sun W., Zhang Z., Zheng Y. (2014). The role of Nrf2-mediated pathway in cardiac remodeling and heart failure. Oxid. Med. Cell Longev..

[137] Suntar I., Cetinkaya S., Panieri E., Saha S., Buttari B., Profumo E., Saso L. (2021). Regulatory Role of Nrf2 Signaling Pathway in Wound Healing Process. Molecules.

[138] Tavleeva M.M., Rasova E.E., Rybak A.V., Belykh E.S., Fefilova E.A., Pnachina E.M., Velegzhaninov I.O. (2023). Dose-Dependent Effect of Mitochondrial Superoxide Dismutase Gene Overexpression on Radioresistance of HEK293T Cells. Int. J. Mol. Sci..

[139] Ridker P.M., Danielson E., Fonseca F.A., Genest J., Gotto A.M., Kastelein J.J., Koenig W., Libby P., Lorenzatti A.J., MacFadyen J.G. (2008). Rosuvastatin to prevent vascular events in men and women with elevated C-reactive protein. N. Engl. J. Med..

[140] Group S.R., Wright J.T., Williamson J.D., Whelton P.K., Snyder J.K., Sink K.M., Rocco M.V., Reboussin D.M., Rahman M., Oparil S. (2015). A Randomized Trial of Intensive versus Standard Blood-Pressure Control. N. Engl. J. Med..

[141] Raber L., Ueki Y., Otsuka T., Losdat S., Haner J.D., Lonborg J., Fahrni G., Iglesias J.F., van Geuns R.J., Ondracek A.S. (2022). Effect of Alirocumab Added to High-Intensity Statin Therapy on Coronary Atherosclerosis in Patients with Acute Myocardial Infarction: The PACMAN-AMI Randomized Clinical Trial. JAMA.

